# LL22NC03-N14H11.1 regulates the m6A modification of MYC and promotes glycolytic activity in hepatocellular carcinoma cells by inhibiting IGF2BP3 ubiquitination degradation

**DOI:** 10.1186/s13046-025-03606-1

**Published:** 2025-12-02

**Authors:** Tingzhuang Yi, Juan He, Meini Pan, Yujie Wang, Cheng Lin, Yulu Ye, Wanlin Yang, Xia Ye, Dengchong Ning, Jinyan Lan, Junlin Huang, Shengkui Tan, Huafu Li, Zhongheng Wei, Cheng Yuan

**Affiliations:** 1 Department of Oncology, Affiliated Hospital of YouJiang Medical University For Nationalities, Baise, Guangxi P. R. China; 2 Guangxi Clinical Medical Research Center for Hepatobiliary Diseases, Baise, Guangxi P. R. China; 3 Department of Emergency, Renmin Hospital, Wuhan University, Wuhan, Hubei P. R. China; 4 Department of Tuberculosis Section One, Puyang Fifth People’s Hospital, Puyang, Henan P. R. China; 5 Affiliated Hospital of YouJiang Medical University For Nationalities, Baise, Guangxi P. R. China; 6 Guangxi Key Laboratory of Environmental Exposomics and Entire Lifecycle Health, Guilin Medical University, Guilin, Guangxi P. R. China; 7 Imperial College London, Sir Alexander Fleming Building, South Kensington Campus, London, UK; 8 Department of Oncology, Yichang Central People’s Hospital and The First College of Clinical Medical Science, China Three Gorges University Yichang, Yichang, Hubei, P. R. China; 9 Tumor Prevention and Treatment Center of Three Gorges University and Cancer Research Institute of Three Gorges University Yichang, Yichang, Hubei P. R. China; 10 Clinical Medical Research Center for Precision Diagnosis and Treatment of Lung Cancer and Management of Advanced Cancer Pain of Hubei Province, Yichang, Hubei P. R. China; 11 Central Laboratory, The First College of Clinical Medical Science, China Three Gorges University & Yichang Central People’s Hospital, Yichang, Hubei P. R. China

**Keywords:** Hepatocellular carcinoma (HCC), Long non-coding RNAs (lncRNAs), LL22NC03-N14H11.1, IGF2BP3, Ubiquitination

## Abstract

**Background:**

Hepatocellular carcinoma (HCC) is the fifth leading cause of cancer-related mortality globally. Long non-coding RNAs (lncRNAs) are increasingly recognized for their pivotal roles in hepatocarcinogenesis. Specifically, the lncRNA LL22NC03-N14H11.1 (hereafter referred to as LL22NC03) has been characterized as a potent oncogenic factor in certain cancers. Therefore, our research aimed to investigate the involvement of LL22NC03 in HCC progression.

**Methods:**

We analyzed the expression of IGF2BP3 in HCC specimens obtained from The Cancer Genome Atlas (TCGA) dataset. Further investigation via RNA pull-down and mass spectrometry analysis identified LL22NC03 as a binding partner of IGF2BP3, with LL22NC03 enhancing the stability of IGF2BP3 by inhibiting TRIM25-mediated ubiquitination. Subsequent in vitro and in vivo experiments were conducted to explore the modulation of LL22NC03 expression, in combination with manipulation of IGF2BP3 levels, which significantly impacted glycolysis, proliferation, migration, and invasiveness of HCC cells.

**Results:**

The study identified LL22NC03 as a promoter of HCC proliferation and migration of HCC cells. LL22NC03 was observed to bind to the ubiquitination site of IGF2BP3, thereby preventing its degradation and enhancing its stability. This interaction ultimately inhibited the degradation of IGF2BP3. Additionally, the interaction between LL22NC03 and IGF2BP3 facilitated the transcription of MYC, leading to the upregulation of glycolytic genes, including HK2, LDHA, GLUT1, PKM2, and PDK1. Finally, IGF2BP3 played a role in stabilizing MYC by recognizing N6-methyladenosine (m6A) modifications.

**Conclusion:**

The LL22NC03-IGF2BP3-MYC regulatory axis is critically involved in the progression of HCC, suggesting its potential as a novel therapeutic target for this malignancy.

**Supplementary Information:**

The online version contains supplementary material available at 10.1186/s13046-025-03606-1.

## Background

Hepatocellular carcinoma (HCC), a malignant tumor typically originating from the liver tissue [1], ranks as the fifth leading cause of cancer-related mortality worldwide [2]. HCC is characterized by its resistance to existing anticancer therapies, and its incidence has steadily increased in recent years. Projections indicate a substantial increase in HCC diagnoses over the next decade, which highlights the urgent need for more effective treatment options [3, 4]. Despite advances in surgery, radiotherapy, chemotherapy, and immunotherapy, survival rates for patients with HCC remain suboptimal, primarily due to the frequent occurrence of tumor recurrence and metastasis [5]. Consequently, elucidating the molecular mechanisms underlying HCC progression is essential for identifying potential therapeutic targets. Given these circumstances, prompt and prioritized identification of key molecules involved in HCC is imperative to improve clinical outcomes.

Long non-coding RNAs (lncRNAs), which are structurally similar to messenger RNAs and range in length from 200 nucleotides to over 100 kilobases, are characterized by their absence of open reading frames, precluding them from encoding proteins [6]. These RNA molecules are predominantly located in both the nucleus and cytoplasm, where they play essential roles in regulating various biological processes, including cell development, proliferation, and apoptosis [7]. The importance of lncRNAs is particularly notable in the context of tumor initiation, progression, and metastasis. Investigating the role of lncRNAs in HCC progression and metastasis is essential for understanding its pathogenesis and developing new diagnostic and therapeutic strategies. Given our preliminary observations that lncRNA LL22NC03-N14H11.1 (hereafter referred to as LL22NC03) may participate in HCC progression [8], yet with its molecular mechanism remaining unclear. Our objective was to define its context-specific mechanism of action in HCC and delineate its downstream pathways and functional consequences.

Insulin-like growth factor-2 mRNA-binding protein 3 (IGF2BP3) is a pivotal RNA binding-protein (RBP) within the IMP (IGF2 mRNA binding-protein) family, along with IMP1 and IMP2 [9]. These proteins are crucial for post-transcriptional regulation of mRNA and influence a range of cellular processes. Due to its frequent overexpression in various cancers, IGF2BP3 has become a significant focus of cancer research, which suggests its potential utility as a biomarker for diverse malignancies [10]. IGF2BP3 overexpression in cancers underscores its importance in cancer biology and its potential value in diagnostic and therapeutic contexts. Elevated levels of IGF2BP3 can promote cancer development and influence disease progression by regulating the cell cycle and apoptosis [11, 12]. Moreover, existing studies suggest that IGF2BP3 exhibits tissue-specific expression and participates in various cancer-related signaling pathways [13–16].

In this study, we aimed to elucidate the role of the LL22NC03 in HCC progression and its underlying molecular mechanisms. Through a series of in vitro and in vivo experiments, we demonstrated that the LL22NC03-IGF2BP3 axis plays a critical role in HCC cell proliferation, migration, and metabolic reprogramming. Our findings not only provide novel insights into the oncogenic functions of LL22NC03 in HCC but also highlight its potential as a therapeutic target for this deadly disease.

## Methods

### Cell culture

The THLE-3 (normal liver), 293 T (human renal epithelial), Huh7, and SK-HEP-1 (HCC) cell lines, which were obtained from the American Type Culture Collection (Manassas, VA, USA), were cultured in Dulbecco’s Modified Eagle Medium (DMEM; Gibco, Rockville, MD, USA) supplemented with 10% fetal bovine serum (FBS; Gibco) and 1% penicillin‒streptomycin (Gibco). All cell lines were maintained at 37 °C in a humidified incubator with 5% CO₂. The medium was changed every three days, and cells were passaged upon reaching 80–90% confluence.

### Reagents and antibodies

The following primary antibodies were used in this study: LDHA (Abcam, ab300637), HK2 (Abcam, ab209847), IGF2BP3 (Abcam, ab177477), MYC (Abcam, ab32072), GAPDH (Abcam, ab8245), m6A (Abcam, ab208577), IgG (Abcam, ab172730), Ki-67 (Affinity Biosciences, AF0198), Myc-Tag (Abclonal, AE070), DYKDDDDK-Tag (Abclonal, AE092), His-Tag (Proteintech, 66,005–1-Ig), HA-Tag (Proteintech, 51,064–2-AP), Ubiquitin (Abclonal, A0162), and EPCAM (Proteintech, 21,050–1-AP). For secondary detection, an HRP-conjugated goat anti-rabbit IgG (H + L) (Abclonal, AS014) was employed. Nuclei were counterstained with DAPI (Abclonal, RM02978). All antibodies were diluted and applied according to the manufacturers’ instructions.

### RNA isolation, reverse transcription, and quantitative real-time PCR (qRT-PCR)

Total cellular RNA was extracted using TRIzol reagent (Invitrogen, Carlsbad, CA, USA) and was reverse transcribed into cDNA via a High-Capacity cDNA Reverse Transcription Kit (Thermo Fisher Scientific, Waltham, MA, USA). Quantitative real-time PCR (RT‒qPCR) analyses were conducted using SYBR Green PCR Master Mix (Takara, Kyoto, Japan) on an ABI Prism 7900HT sequence detector (Applied Biosystems, Foster City, CA, USA). Relative gene expression was calculated using the 2^−ΔΔ^CT method, and GAPDH and U6 served as internal controls for normalization. All primer sequences are listed in Table 1 .

**Table 1:** The sequences of PCR primers

Gene name	Sequence
LL22NC03-N14H11.1	Forward:5′-GAGTCTGGGGATCAGCATCG −3′
	Reverse:5′-TCCAGGGGGCTGGATAATGA −3′
IGF2BP3	Forward 5′-TATATCGGAAACCTCAGCGAGA-3′
	Reverse 5′-GGACCGAGTGCTCAACTTCT-3′
MYC#1	Forward: 5′-CGTCCTCGGATTCTCTGCTC-3’
	Reverse: 5′- GCTGGTGCATTTTCGGTTGT-3′
MYC#2	Forward: 5′-TTGCGGAAACGACGAGAACA-3'
	Reverse: 5′-TCATAGGTGATTGCTCAGGACA-3'
GAPDH	Forward 5′-CTGGGCTACACTGAGCACC-3′
	Reverse 5′-AAGTGGTCGTTGAGGGCAATG-3′
U6	Forward 5′-GCAGACCGTTCGTCAACCTA-3′
	Reverse 5′-AATTCTGTTTGCGGTGCGTC-3′
LDHA	Forward: 5’- AGGCTATTCTTGGGCAACCC −3′
	Reverse: 5′-TGAGTAGACATCCACCAAGGTT −3′
HK2	Foward: 5′-GATTGCCTCGCATCTGCTTG-3′
	Reverse: 5′-CCAAAGCACACGGAAGTTGG-3′
GLUT1	Forward: 5′-ATTGGCTCCGGTATCGTCAAC-3′
	Reverse: 5′-GCTCAGATAGGACATCCAGGGTA −3′
PKM2	Foward: 5′-CTCTTCAGAAGTCCCCAGCG-3′
	Reverse:5′- GCTCGACCCCAAACTTCAGA-3′
PDK1	Foward:5′-GGCCAGGTGGACTTCTACG-3′
	Reverse:5′- ACATTCTGGCTGGTGACAGG-3′

**Table 2:** The interference sequences were presented as follows

Gene name	Sequence
sh-NC	5′-CCGGGGATTCCTATCCCCTGTCAATCTCGAGTTTGACAGGGGAAACCAATCGTTTTTG-3′
sh-LL22NC03-N14H11.1#1	5′-CCGGGCACTGGTATGGGCTGTCTATCTCGAGATAGACAGCCCATACCAGTGCTTTTTG-3′
sh-LL22NC03-N14H11.1#2	5′-CCGGGGAATCAGGCCTCCCAAATTTCTCGAGAAATTTGGGAGGCCTGATTCCTTTTTG-3′
sh-NC	CCGGTTCTCCGAACGTGTCACGTTTTTCAAGAGAAAACGTGACACGTTCGGAGAATTTTTTGGTACC
sh-IGF2BP3	CCGGATTATGAGCTAAAATCTTCAATTCAAGAGATTGAAGATTTTAGCTCATAATTTTTTTGGTACC
si-NC	5′-TTCTCCGAACGTGTCACGTTT-3
si-METTL3	5′- GGUUGGUGUCAAAGGAAAUTT −3′
si-METTL14	5’-UCAGUUUAGGAUAUUCUUCAA-3’
si-WTAP	5’-ACUGUUACAAUUUCCAAAGCG-3’

### Lentiviral packaging and cell transfection

Short hairpin RNAs (shRNAs) targeting LL22NC03 and IGF2BP3, along with their corresponding negative controls, as well as overexpression plasmids for LL22NC03 and IGF2BP3 and their respective empty vector controls, were all synthesized by GeneChem (Shanghai, China). For routine transfection of cell lines, SK-HEP-1 or Huh7 cells were seeded in 6-well plates at a density of 1 × 10^6^ cells per well. Upon reaching 70–80% confluence, the cells were transfected using either Lipofectamine 2000 (Invitrogen, Carlsbad, CA, USA) or X-tremeGENE transfection reagent (Thermo Fisher Scientific, USA), strictly following the manufacturers’ protocols. All transfection experiments were independently performed in triplicate. The specific sequences of the shRNAs used are listed in Table 2.

### Lentiviral infection of organoids

To establish HCC organoid models with stable gene knockdown or overexpression, we employed a lentiviral system for genetic manipulation. First, the respective plasmids (overexpression or shRNA) and their control plasmids were co-transfected with the packaging plasmids psPAX2 and pMD2.G into HEK293T cells for lentivirus production. Viral supernatants were collected 48 to 72 h post-transfection and concentrated via ultracentrifugation.

For organoid infection, the organoids were initially treated with a Cell Recovery Solution for 1 h at 4 °C to dissolve the Matrigel. After centrifugation, the harvested organoids were dissociated into single-cell suspensions using TrypLE (Gibco). The single cells were resuspended in transduction medium supplemented with TransDUX MAX (Takara Bio) enhancer and seeded into ultra-low attachment 24-well plates. An appropriate volume of the concentrated lentiviral supernatant was then added to achieve a multiplicity of infection (MOI) of 10. The plates were sealed with Parafilm and centrifuged at 600 × g for 1 h at 32 °C, followed by incubation at 37 °C for 4–6 h. Following transduction, the cells were collected, centrifuged, and re-embedded in Matrigel for continued culture. Forty-eight hours post-transduction, puromycin was added to the culture medium at a final concentration of 2 μg/mL for selection. The selection process was maintained for 4 days or until complete death of the non-transduced control organoids was observed. Stable knockdown or overexpression of the target genes in the selected organoid populations was confirmed by qPCR.

### Site-directed mutagenesis plasmid construction

Based on molecular docking analysis, we identified four potential key binding residues (Asp294, Gly296, Ile312, and Thr328) on the IGF2BP3 protein for site-directed mutagenesis. By individually mutating each of these sites to arginine (Arg), we successfully generated four corresponding mutant plasmids, designated as D294R, G296R, I312R, and T328R. All site-directed mutagenesis plasmids were custom-synthesized by YouBio (Changsha, Hunan, China).

### Proliferation assays

After transfection, SK-HEP-1 and Huh7 cells were seeded in 96-well plates at a density of 5 × 10^3^ cells per well in complete medium. Cell viability was assessed by incubation with 10 µL of CCK-8 solution (Dojindo, Kumamoto, Japan) per well for 2 h. The optical density at 450 nm was measured at 0, 24, 48, 72, and 96 h intervals in a microplate reader (Dynex Technologies, West Sussex, UK). Each viability assay was conducted in triplicate for consistency. Concurrently, a separate set of cells was subjected to an EdU incorporation assay. After treatment with EdU and incubation under standard conditions, the cells were fixed, permeabilized, and blocked before staining. Fluorescence microscopy was used to visualize and quantify the EdU-labeled DNA, which provided insights into DNA synthesis activity.

### Wound healing

Posttransfection, SK-HEP-1 and Huh7 cells were plated at a density of 1 × 10^6^ cells/well in 6-well plates. Upon reaching 80–90% confluence, a wound was generated using a 200 µL filter tip. The cells were then incubated in serum-free medium at 37 °C for 24 h to facilitate wound healing. Wound closure was documented at 0 and 24 h via an Olympus microscope (Tokyo, Japan). This wound healing assay was meticulously repeated in triplicate to ensure reliable and reproducible data.

### Invasion assay

Cell invasion assays were conducted using 24-well plates containing Transwell inserts (8-μm pore size; Corning Incorporated, Big Flats, NY, USA) coated with Matrigel (BD Biosciences) to simulate extracellular matrix conditions. Transfected cells (2 × 10^4^) in serum-free medium were seeded in the upper chamber, while the lower chamber contained complete medium supplemented with 10% FBS as a chemoattractant. After 24 h, the cells that had invaded through the membrane were fixed in 4% formaldehyde, stained with crystal violet, and counted under an optical microscope (Thermo Fisher). This invasion assay was rigorously replicated three times to ensure precise reproducibility so that this assay met the stringent standards of scientific research.

### Western blotting

Total cellular proteins were extracted in RIPA lysis buffer and separated on SDS‒polyacrylamide gels. The proteins were then transferred to PVDF membranes (Bio-Rad Laboratories, Hercules, CA, USA), which were blocked with 5% nonfat milk and incubated overnight at 4 °C with primary antibodies. After they were washed in TBST, the membranes were incubated with secondary antibodies at room temperature for 2 h. The protein bands were visualized via enhanced chemiluminescence (ECL) (Pierce, Rockford, IL, USA) and quantified with ImageJ software (National Institutes of Health, USA). Western blotting was performed in triplicate to ensure robust reproducibility and adherence to rigorous scientific experimentation and documentation standards.

### Immunohistochemistry

The tumor tissues from the xenograft assays were fixed in 4% paraformaldehyde, dehydrated, and then embedded in paraffin. Sections (4 mm thick) were prepared from these paraffin-embedded tissues and then deparaffinized. These sections were incubated overnight at 4 °C with primary antibodies against MYC (Abcam, ab32072), LDHA (Abcam, ab300637), HK2 (Abcam, ab209847), and IGF2BP3 (Abcam, ab177477), followed by a 30-min incubation at 37 °C with a biotinylated secondary antibody. To ensure accuracy and reproducibility, this immunohistochemical analysis was meticulously performed in triplicate, adhering to scientific research and reporting standards.

### RNA stability assay

To evaluate mRNA stability, cells were treated with 1 µg/mL Actinomycin D (ActD). RNA was isolated at subsequent intervals, after which qRT‒PCR was conducted. The decay of mRNA following ActD treatment was plotted to determine the mRNA half-life. This method allows for accurate assessment of mRNA stability.

### RNA pull-down assay

The RNA pull-down assay utilized the Pierce Magnetic RNA–Protein Pull-Down Kit (Thermo Fisher Scientific, Waltham, MA, USA). Protein lysates from SK-HEP-1 and Huh7 cells were extracted in RIPA lysis buffer. These lysates were then incubated with biotinylated RNA probes (LL22NC03 with or without biotin). Streptavidin-coated magnetic beads (Invitrogen) were used to capture RNA–protein complexes, which were subsequently analyzed by Western blotting.

### RNA immunoprecipitation

For RNA immunoprecipitation (RIP) assays, the Magna RIP™ RNA-Binding Protein Immunoprecipitation Kit from Millipore (CA, USA) was used. The cell lysates, which were prepared using the RIP lysis buffer provided with the kit, were incubated with magnetic beads conjugated to an anti-IGF2BP3 antibody integrated within the RIP buffer. An anti-IgG antibody served as the negative control, while the interaction between U1 and SNRNP70 was used as the positive control. Following incubation, proteinase K was added to the mixture, and the resulting precipitates were analyzed by RT-qPCR.

### Fluorescence in situ hybridization

RNA fluorescence in situ hybridization (FISH) probes targeting LL22NC03 and IGF2BP3 were custom-designed and synthesized by General Biosystems Co. Ltd. (Anhui, China). For the FISH assay, cell samples were initially fixed in 4% formaldehyde for 15 min, followed by washing with phosphate-buffered saline (PBS). The subsequent steps involved pepsin treatment and cell dehydration. The air-dried samples were then incubated with the respective FISH probes in hybridization buffer. Nuclei were stained with 4′,6-diamidino-2-phenylindole (DAPI), and the cells were imaged under a laser scanning confocal microscope (Zeiss, Jena, Germany).

### Immunoprecipitation

Transfected cells were lysed in immunoprecipitation (IP) lysis buffer, followed by centrifugation to collect the cell lysates. These lysates were then incubated overnight with anti-IGF2BP3 antibody (Abcam, ab177477) at 4 °C with constant agitation. Normal immunoglobulin G (IgG) (Abcam, ab172730) served as the negative control. The antigen–antibody complexes were subsequently incubated with protein A-Sepharose beads. After three washes with IP lysis buffer, the complexes were eluted and analyzed by immunoblotting (Western blot).

### Molecular docking analysis

Rigid protein–protein docking was performed between TRIM25 and IGF2BP3 to investigate their interactions using GRAMM‐X (http://gramm.compbio.ku.edu/). The protein structural domains of TRIM25 and IGF2BP3 were obtained from the Protein Data Bank (PDB) database (http://www.rcsb.org/).The interaction surfaces of the predicted complexes were analyzed online by PDBePISA (https://www.ebi.ac.uk/pdbe/pisa/). Finally, conformational mapping and docking region analysis were performed using PyMOL (https://pymol.org/) and LigPlus (https://www.ebi.ac.uk/thornton-srv/software/LigPlus/).

### Ubiquitination assay

Transfected cells were lysed in preheated lysis buffer containing 1% SDS and 10 mM N-ethylmaleimide (Sigma-Aldrich), followed by boiling at 100 °C for 10 min. The resulting cell lysates were then diluted with SDS-free cell lysis buffer. This mixture was incubated with anti-Flag-M2 agarose for immunoprecipitation. The immunoprecipitates were analyzed by Western blotting.

### Glycolytic activity

Glycolytic activity in Huh7 hepatocellular carcinoma cells was assessed via a Seahorse XF24 analyzer (Seahorse Bioscience). The cells (100,000 per well) were cultured overnight on fibronectin-gelatin-coated plates and equilibrated in nonbuffered medium in a non-CO_2_ incubator for 30 min before the assay. The extracellular acidification rate (ECAR) was measured at 4-min intervals for five cycles, with activators/inhibitors (10 mM glucose, 1 µM oligomycin, and 100 mM 2-DG from Sigma) added after each cycle. The protein concentration was then determined via a Bio-Rad protein assay. ECAR values were normalized to protein levels. The cells were treated with OxPAPC for 24 h before glycolysis was measured, and the glycolytic rate and capacity were determined after the addition of glucose and oligomycin, respectively.

### MeRIP-qPCR assay for quantifying m6a modifications in MYC mRNA

m6A modifications in MYC mRNA across various cell lines, including HCC cells, were quantified using methylated RNA immunoprecipitation (Me-RIP). Total RNA was extracted using TRIzol, which was followed by Me-RIP with 3 μg of anti-m6A antibody (Abcam, ab208577) coupled to protein A/G beads and an incubation overnight at 4 °C. Subsequently, 100 μg of RNA was incubated with this complex, and m6A-modified RNA was eluted with N6-methyladenosine 5'-monophosphate sodium salt (Sigma-Aldrich, M2780). The purification process involved phenol‒chloroform extraction followed by ethanol precipitation. For RT-qPCR, 10 ng of purified RNA was subjected to Superscript III reverse transcriptase with MYC mRNA-specific primers. m6A enrichment was calculated via the 2^−ΔΔ^Ct method, and the immunoprecipitated RNA was compared with the input RNA. Statistical significance was assessed using Student’s t test, with *p* < 0.05 considered statistically significant. This protocol ensures accurate quantification of m6A modifications in MYC mRNA, adhering to scientific reporting standards.

### Isolation and culture of tumor organoids

Fresh tumor tissues obtained from hepatocellular carcinoma patients were fragmented, washed with PBS, and digested using a tumor tissue digestion solution (K601003, BioGenous Technologies, Shanghai, China). The resulting suspension was filtered through a 100 μm filter to remove tissue debris. Cells were resuspended in Advanced DMEM/F12 medium (Gibco) and mixed with an equal volume of Matrigel in 24-well plates. Accuroid Organoid Medium—Hepatocellular Carcinoma (Accurate International Biotechnology) was then added to cover the Matrigel, and cells were cultured at 37 °C in a CO₂ cell incubator. The medium was replenished every 4 days, and organoids were passaged every 1–4 weeks. The study was approved by the Medical Ethics Committee of Yichang Central People’s Hospital (IRB No. 2025–474-01).

### Patient-Derived Organoid Subcutaneous Xenograft (PDO-SC) mouse model

Eight-week-old BALB/c nude mice (SLAC Laboratory Animal Co., Ltd., Shanghai) were housed in standard barrier facilities at 20–26℃ under a 12 h light/dark cycle, with free access to autoclaved water and standard rodent chow. Mice were allocated into two groups: Myc-overexpression group and Myc-overexpression + 10058-F4 intervention group. Organoids were transduced with lentiviral vectors (RiboBio Co., Ltd., Guangzhou) for Myc overexpression at an MOI of 10, supplemented with 8 μg/ml polybrene. After 24 h incubation, the culture medium was replaced, followed by an additional 48–72 h of cultivation. Organoids were dissociated into single cells using TrypLE, and 1 × 10^5^ cells were mixed with an equal volume of 50% Matrigel/50% Hank’s Balanced Salt Solution (HBSS) for subcutaneous inoculation into the dorsal region of nude mice (6 mice per group). The Myc-overexpression + 10058-F4 group received intraperitoneal injections of 10058-F4 at a dose of 25 mg/kg, once daily for 5 consecutive days, with a 2-day interval before repeating the course for 2 weeks. The control group received an equivalent volume of vehicle. Mice were euthanized 1 month post-inoculation, and tumors were excised, photographed, and measured using the formula: 0.5 × width^2^ × length (mm^3^). All procedures complied with the guidelines of the Institutional Animal Care and Use Committee (IACUC), and the protocol was approved by the Ethics Committee for Animal Research of the Affiliated Hospital of Youjiang Medical University for Nationalities.

### Xenograft tumor model of tumor cells

Four-week-old male BALB/c nude mice were purchased from Shi Laike Company (Shanghi, China) and maintained in SPF-grade, pathogen-free animal lab. Tumor xenograft assay was performed via injecting nude mice subcutaneously with 1 × 10^6^ SK-HEP-1 cells transfected with sh-LL22NC03, sh-LL22NC03 + sh-IGF2BP3, or sh-NC (mice were randomly divided into three groups). Tumor volume was recorded every 4 days. Thirty days later, mice were killed by cervical dislocation and tumors were weighted. Animal studies were approved by the Animal Ethics Committee of Affiliated Hospital of Youjiang Medical College For Nationalities The work has been reported in line with the ARRIVE criteria [17].

### Statistical analysis

Data following a normal distribution from assays conducted in triplicate are presented as the means ± SDs. Statistical analysis was conducted via SPSS V.27.0 (SPSS, Chicago, IL, USA) or Prism 6 (GraphPad Software, San Diego, CA, USA). Overall survival was plotted using the Kaplan–Meier method and compared via the log-rank test. Gene expression correlations were analyzed via Pearson’s correlation. Student’s t test or analysis of variance (ANOVA) was used to determine significant differences between groups, with *P* < 0.05 considered statistically significant.

## Results

### LL22NC03 promotes the proliferation, migration, and invasion of hepatocellular carcinoma cells

**Fig. 1:**
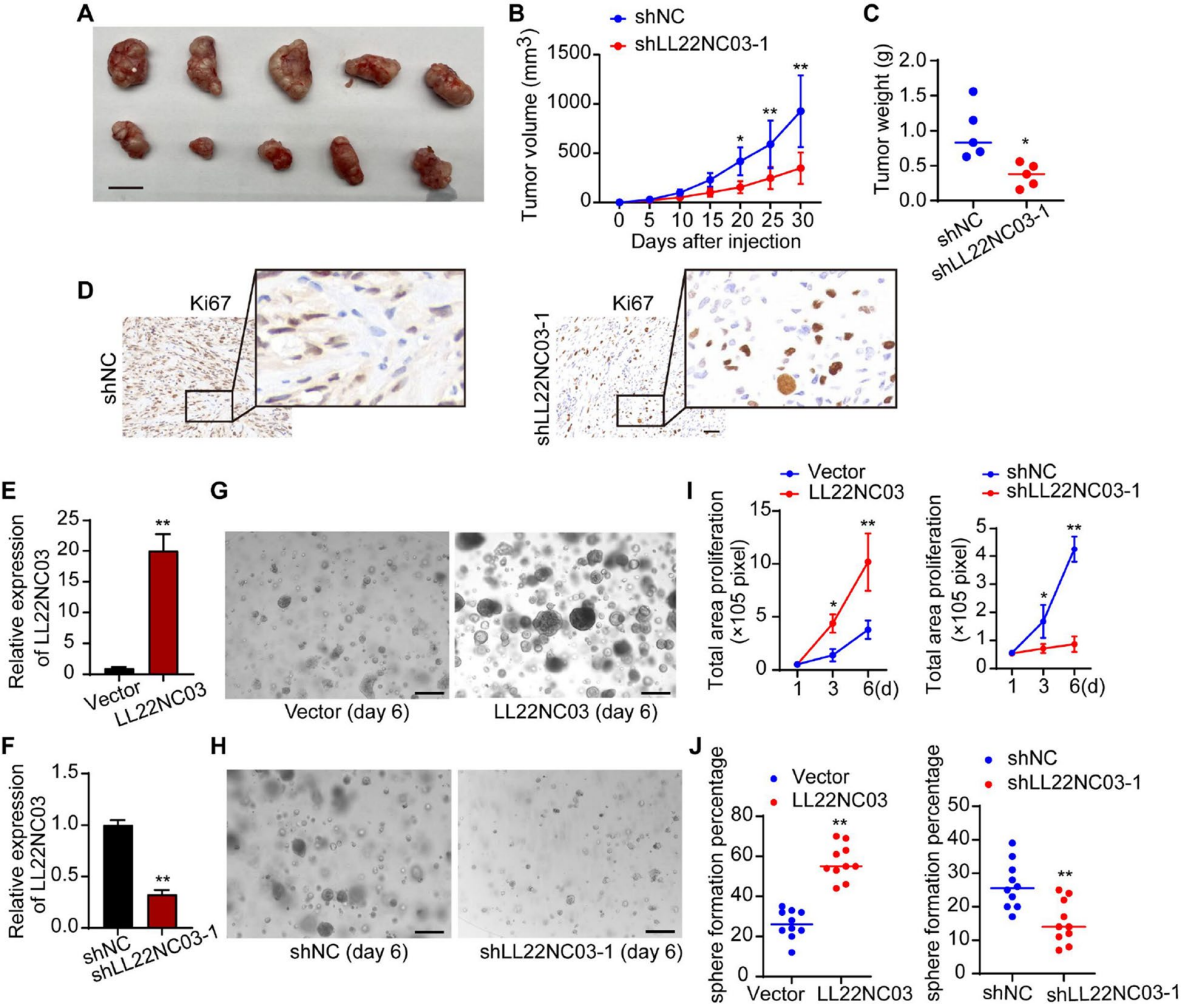
LL22NC03 promotes the progression of subcutaneous xenograft tumors and enhances the growth and formation of HCC organoids. **A** Representative images of subcutaneous xenograft tumors formed by Huh7 cells in nude mice. Scale bar = 10 mm. **B **Growth curves of xenograft tumor volumes in mice. **C **Quantitative analysis of xenograft tumor weights in mice. **D** Representative images of Ki67 immunohistochemical staining in xenograft tumor tissues. Scale bar = 50 μm. **E** qRT-PCR analysis of LL22NC03 overexpression efficiency in HCC organoids transfected with lentivirus. **F** qRT-PCR analysis of LL22NC03 knockdown efficiency in HCC organoids transfected with lentivirus. **G** Representative images of HCC organoid spheres. Compared with the control group, the sphere area of LL22NC03-overexpressing HCC organoids was increased. Scale bar = 100 μm. **H** Representative images of HCC organoid spheres. Compared with the control group, the sphere area of LL22NC03-knockdown HCC organoids was reduced. Scale bar = 100 μm. **I** Quantitative analysis of total proliferative area of HCC organoid spheres. The sphere area was significantly larger in the LL22NC03 overexpression group and significantly smaller in the LL22NC03 knockdown group compared with the control group. **J** Quantitative analysis of HCC organoid formation efficiency. The organoid formation efficiency was significantly higher in the LL22NC03 overexpression group and significantly lower in the LL22NC03 knockdown group compared with the control group. (^*^P < 0.05, ^**^P < 0.01, n = 3)

To investigate the clinical relevance of LL22NC03 in HCC, we first examined its expression level in clinical samples. RT-qPCR analysis revealed that LL22NC03 was significantly upregulated in HCC tissues compared to adjacent non-tumorous tissues (Supplementary Fig. 1 A). To explore the functional role of LL22NC03 in HCC, we established an LL22NC03 overexpression model using a lentiviral vector, with overexpression efficiency confirmed by RT-qPCR (Supplementary Fig. 1B). Consistent with previous findings indicating high expression of LL22NC03 in hepatic epithelial cells and various HCC cell lines, particularly in Huh7 and SK-HEP-1 cells, and its further elevation in advanced and metastatic HCC patient tissues [8]. We selected Huh7 and SK-HEP-1 cell lines for subsequent functional studies. Given this expression profile, we hypothesized that LL22NC03 might promote malignant phenotypes in HCC cells. CCK-8 and EdU assays demonstrated that LL22NC03 overexpression significantly enhanced the proliferative capacity of HCC cells (Supplementary Fig. 1 C, D). Furthermore, Transwell and wound healing assays indicated that LL22NC03 overexpression markedly increased the migratory and invasive abilities of HCC cells (Supplementary Fig. 1E, F). In vivo subcutaneous xenograft models showed that knockdown of LL22NC03 significantly suppressed tumor growth, as evidenced by reduced tumor volume and weight (Fig. 1A-C), accompanied by a decrease in the proportion of Ki67-positive cells in the tumor tissues (Fig. 1D). Collectively, these results demonstrate that LL22NC03 promotes HCC cell proliferation, migration, and invasion in vitro and enhances tumor growth in vivo.

### LL22NC03 enhances the growth and formation of HCC organoids

To further validate these findings in a model that more closely recapitulates the in vivo tumor microenvironment, we successfully established patient-derived HCC organoids. Efficient overexpression or knockdown of LL22NC03 in these organoids, achieved via lentiviral transduction, was confirmed by RT-qPCR (Fig. 1E, F). Functional assessments revealed that LL22NC03 overexpression significantly increased organoid sphere size (Fig. 1G, I) and elevated the formation efficiency from 26 to 57% (Fig. 1J). Conversely, LL22NC03 knockdown led to a reduction in sphere size (Fig. 1H, I) and decreased the formation efficiency from 28 to 16% (Fig. 1J). These results demonstrate that LL22NC03 is critical for the growth and formation of HCC organoids. To confirm the reliability of this organoid model for mechanistic studies, we further verified its histological similarity to the primary tumors. Hematoxylin and eosin (H&E) staining showed that the organoids recapitulated the typical architectural features of the original HCC tissues (Supplementary Fig. 2 A). Given that the epithelial cell adhesion molecule (EpCAM) is a well-established biomarker for HCC, particularly in subtypes with stem-like properties or high aggressiveness [18, 19], we assessed its expression by immunofluorescence. The results demonstrated that the expression pattern of EpCAM in the organoids closely mirrored that in their corresponding primary tumors (Supplementary Fig. 2B). Together, these data indicate that our established HCC organoids reliably model the histomorphology and molecular phenotypes of the primary tumors.

### LL22NC03 binds to IGF2BP3

**Fig. 2:**
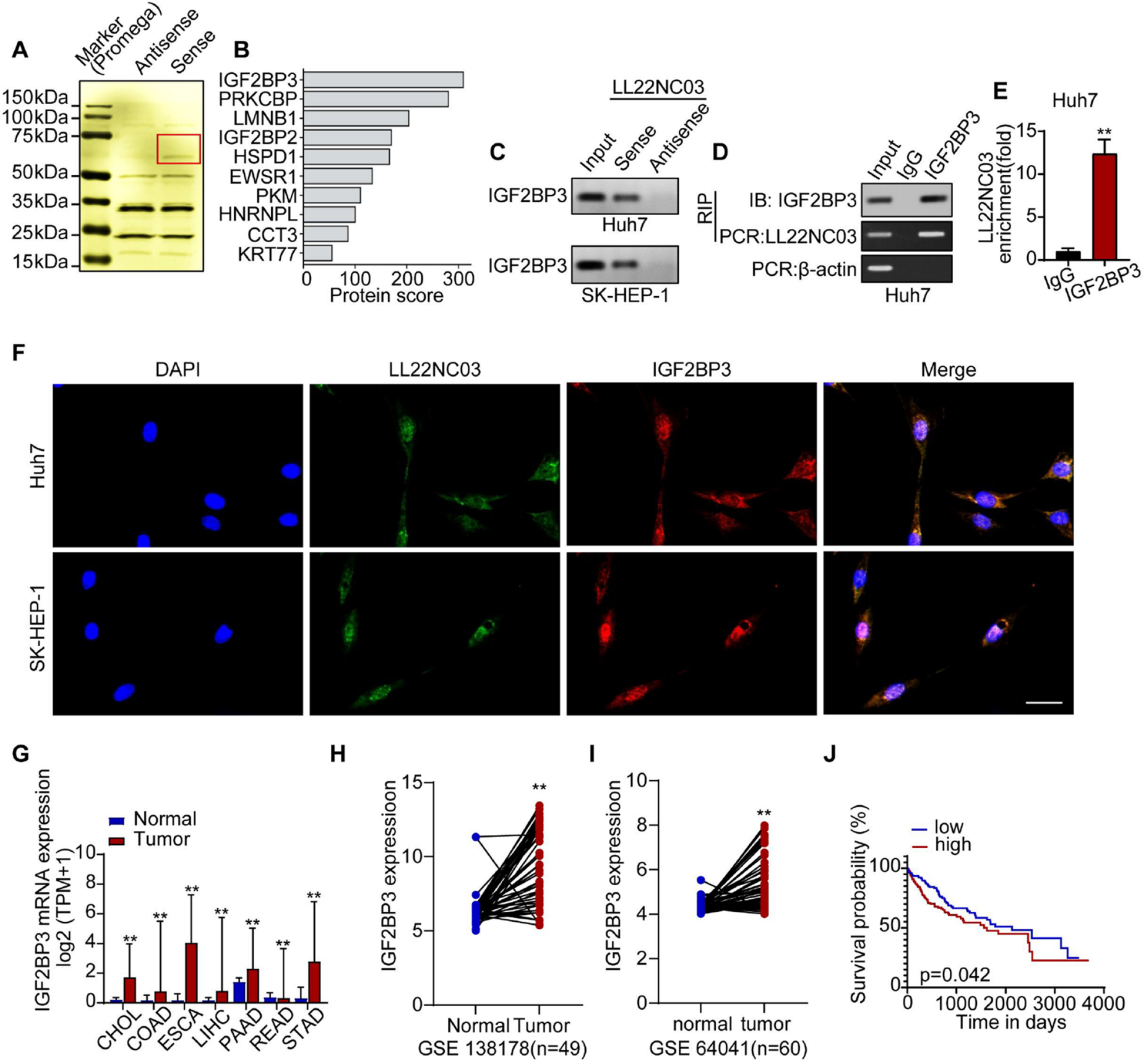
LL22NC03 binds directly to the RBP IGF2BP3. **A** LL22NC03-binding proteins were directly extracted via RNA pull-down. **B** The top 10 proteins obtained from RNA pull-down and mass spectrometry analysis. **C** Downregulation of IGF2BP3 with biotin-labeled LLN22C03 sense (S) but not antisense LLN22C03 (AS) RNA in the indicated cells. **D** and **E** RIP analysis using an anti-IGF2BP3 antibody and extract from Huh7 cells. Relative enrichment indicates the level of RNA associated with the indicated proteins after immunoprecipitation with an anti-IGF2BP3 antibody relative to that after immunoprecipitation with an IgG antibody. **F** Immunofluorescence showed colocalization of IGF2BP3 (red) and LL22NC03 (green). Nuclei were stained with DAPI (blue). Scale bar = 30 μm. **G** The expression of IGF2BP3 in different gastrointestinal tumors and adjacent normal tissues in the TCGA database. **H** Expression of IGF2BP3 in HCC samples from the GSE138178 dataset (*n* = 49). **I** Expression of IGF2BP3 in HCC samples from the GSE64041 dataset (*n* = 60). **J** Kaplan‒Meier analysis of OS curves of HCC patients with low (*n* = 173) or high (*n* = 173) IGF2BP3 expression (log-rank test). (^**^P < 0.01, ^***^P < 0.001)

To elucidate the molecular mechanism by which LL22NC03 drives malignant progression in HCC, we employed a biotin-labeled RNA pull-down assay to identify its interacting proteins. Silver staining revealed a distinct protein band specifically enriched in the LL22NC03 sense group compared to the antisense control, migrating at approximately 50–75 kDa (Fig. 2A). Mass spectrometric analysis identified IGF2BP3 as the top enriched candidate among the pulled-down proteins (Fig. 2B). Given its established role as a key RNA-binding protein involved in post-transcriptional regulation, IGF2BP3 was selected for further validation. Both RNA pull-down and RIP assays confirmed the specific interaction between LL22NC03 and IGF2BP3 (Fig. 2C–E). Furthermore, immunofluorescence staining demonstrated clear cytoplasmic co-localization of LL22NC03 and IGF2BP3 in HCC cells (Fig. 2F). Collectively, these results demonstrate that LL22NC03 directly binds to IGF2BP3 in HCC cells.

To evaluate the clinical relevance of IGF2BP3 in HCC, we interrogated multiple public databases. RNA-seq data from The Cancer Genome Atlas (TCGA) revealed that significantly higher IGF2BP3 expression in gastrointestinal tumors and HCC cell lines compared to corresponding normal tissues or cells (Fig. 2G). Consistent with this, analysis of the GSE138178 and GSE64041 datasets consistently revealed higher IGF2BP3 expression in HCC tissues than in normal liver tissues (Fig. 2H, I). To experimentally validate these findings, we collected 10 paired clinical HCC samples and confirmed by RT-qPCR that IGF2BP3 was significantly upregulated in tumor tissues compared to adjacent non-tumor tissues (Supplementary Fig. 3 A). Furthermore, survival analysis using the Kaplan–Meier Plotter database demonstrated that high IGF2BP3 expression was significantly associated with shorter overall survival in HCC patients (Fig. 2J). Collectively, these findings suggest that IGF2BP3 acts as an oncogenic factor in HCC and serves as a prognostic marker.

### LL22NC03 regulates the stability of the IGF2BP3 protein

**Fig. 3:**
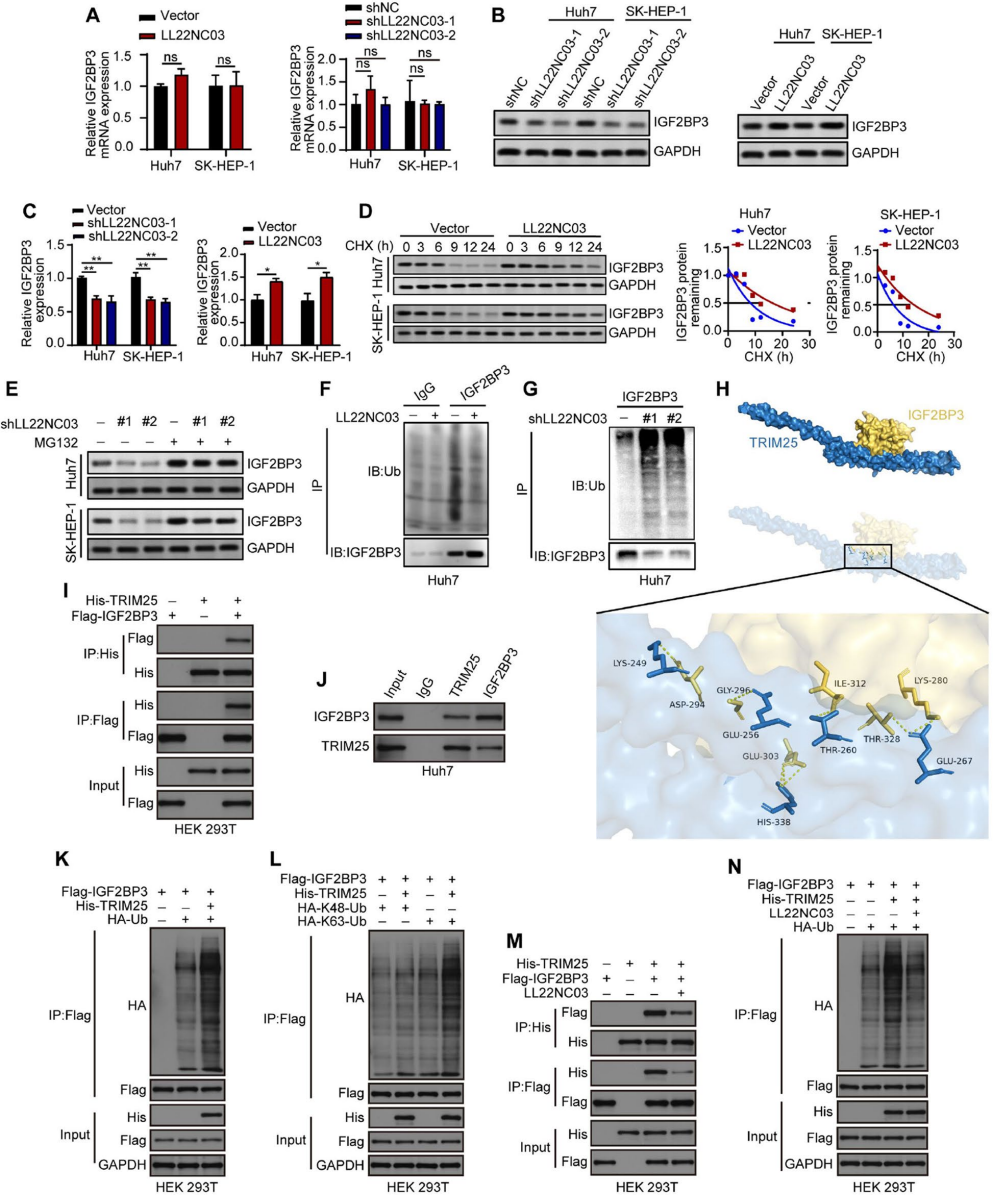
LL22NC03 regulates IGF2BP3 protein stability by inhibiting the interaction between TRIM25 and IGF2BP3. **A** qRT-PCR analysis of IGF2BP3 mRNA levels in HCC cells with LL22NC03 overexpression. **B** and **C** Western blot analysis of IGF2BP3 protein levels in HCC cells with LL22NC03 overexpression or knockdown. **D** Western blot analysis of IGF2BP3 levels in HCC cells (with/without LL22NC03 overexpression) treated with 20 µg/ml CHX for indicated times. **E** Western blot analysis of IGF2BP3 levels in LL22NC03-knockdown (sh-1, sh-2) or control HCC cells treated with/without 5 µM MG132 for 12 h. **F** Co-IP assay of IGF2BP3 ubiquitination in Huh7 cells with LL22NC03 overexpression. **G** Co-IP assay of IGF2BP3 ubiquitination in Huh7 cells with LL22NC03 knockdown (sh-1, sh-2). **H** Complex model of IGF2BP3 and TRIM25 constructed by PYMOL. **I** Co-IP assay for exogenous interaction between TRIM25 (HA-tagged) and IGF2BP3 (Flag-tagged) in HEK293T cells. **J** Co-IP assay for endogenous interaction between TRIM25 and IGF2BP3. **K** Co-IP assay of IGF2BP3 (Flag-tagged) ubiquitination in HEK293T cells co-transfected with HA-Ub and His-TRIM25 (with/without), treated with 5 µM MG132 for 12 h. **L** Co-IP assay of IGF2BP3 (Flag-tagged) ubiquitination (K48/K63-linked) in HEK293T cells co-transfected with HA-Ub (K48/K63) and His-TRIM25 (with/without), treated with 5 µM MG132 for 12 h.** M** Co-IP assay for TRIM25-IGF2BP3 interaction in HEK293T cells co-transfected with Flag-IGF2BP3, His-TRIM25 and LL22NC03 (overexpression). IgG as negative control. **N** Co-IP assay of IGF2BP3 ubiquitination in HEK293T cells co-transfected with Flag-IGF2BP3, His-TRIM25 and LL22NC03 (overexpression). (*P < 0.05, **P < 0.01, ns: not significant; *n* = 3)

To assess the impact of LL22NC03 on the interaction with IGF2BP3, we analyzed the mRNA and protein levels of IGF2BP3 following manipulation of LL22NC03 expression. We found that LL22NC03 did not change IGF2BP3 mRNA levels in HCC cell lines (Fig. 3A), whereas IGF2BP3 protein decreased with LL22NC03 knockdown and increased with LL22NC03 overexpression (Fig. 3B, C). Additionally, we explored the possibility that LL22NC03 may increase IGF2BP3 protein levels by inhibiting its degradation. To investigate the degradation pattern of IGF2BP3, the protein synthesis inhibitor cycloheximide (CHX) was used to determine the half-life of IGF2BP3 following LL22NC03 overexpression in HCC cell lines. Our results indicated that the half-life of IGF2BP3 was extended upon LL22NC03 overexpression (Fig. 3D). Furthermore, the impact of LL22NC03 on IGF2BP3 protein levels was reversed by the proteasome inhibitor MG132 (Fig. 3E). Moreover, the results from the ubiquitination assay suggested that LL22NC03 overexpression may decrease the ubiquitination level of IGF2BP3, providing a protective effect against degradation pathways (Fig. 3F). Conversely, LL22NC03 knockdown increased IGF2BP3 ubiquitination and promoted its degradation (Fig. 3G). Collectively, these findings indicated that LL22NC03 may play a role in stabilizing IGF2BP3 at the protein level, potentially through the modulation of the ubiquitin–proteasome pathway, thus inhibiting proteasome-mediated ubiquitination and subsequent degradation.

### LL22NC03 stabilizes IGF2BP3 via inhibiting TRIM25-induced ubiquitination

Based on the aforementioned experimental results, our data indicate that LL22NC03 modulates IGF2BP3 levels via the ubiquitin pathway. Since TRIM25 has been reported as an E3 ubiquitin ligase for IGF2BP3 [20, 21], we evaluated whether TRIM25 regulated IGF2BP3 expression in HCC cells. Subsequently, we used the method of rigid protein–protein docking to predict the potential interaction domains of IGF2BP3 and TRIM25. The 3D spatial structures of these proteins were obtained from PDB databases, and the most likely complex model of IGF2BP3 (PDB ID:6GQ1) and TRIM25 (PDB ID:4LTB) binding was predicted using the GRAMM online protein docking tool. Interface analysis with PDBePISA indicated a favorable (low) binding free energy for the IGF2BP3-TRIM25 complex, consistent with a stable interaction. As shown in (Fig. 3H), IGF2BP3and TRIM25 formed hydrogen bonds between residues GLY296-GLU256 and ILE312-THR260, revealing that proteins IGF2BP3 and TRIM25 formed a stable protein docking model. LigPlus was used to map the “eyelash figure” of protein–protein interactions, and PyMOL was used to map the electrostatic potential energy (Supplementary Fig. 4 A,B), showing the potential binding domains between IGF2BP3 and TRIM25. Next, Co-IP assays confirmed that TRIM25 interacts with IGF2BP3 both endogenously and exogenously in cells (Fig. 3I, J). Previous studies have confirmed that the TRIM25 protein, as an E3 ubiquitin ligase, can catalyze the formation of two classic types of ubiquitin chains, K48-linked and K63-linked, which mediate distinct biological effects of substrate proteins, respectively [22–26]. To clarify the specific type of ubiquitin chain involved in TRIM25-mediated ubiquitination of IGF2BP3, we performed ubiquitination Co-IP assays to detect the modification levels of K48-linked and K63-linked ubiquitin chains on IGF2BP3 by TRIM25, respectively. The results showed that TRIM25 predominantly enhanced the K63-linked polyubiquitination of IGF2BP3, rather than the K48-linked ubiquitination (Fig. 3L). This finding suggests that TRIM25 may regulate the stability of IGF2BP3 through K63-linked ubiquitination.

To investigate whether LL22NC03 influences the interaction between TRIM25 and IGF2BP3, we co-expressed Flag-IGF2BP3 and His-TRIM25 in HEK293T cells in the presence or absence of LL22NC03 overexpression. The results showed that LL22NC03 overexpression markedly weakened the TRIM25-IGF2BP3 interaction (Fig. 3M) and reduced TRIM25-mediated ubiquitination of IGF2BP3 (Fig. 3N). These findings suggest that LL22NC03 stabilizes IGF2BP3 by interfering with its binding to TRIM25, thereby suppressing ubiquitination and subsequent proteasomal degradation. To further identify the key binding sites underlying the interaction between TRIM25 and IGF2BP3, we first screened four potential critical binding residues on IGF2BP3 through molecular docking analysis. Subsequently, we constructed site-directed mutant plasmids targeting each of these residues, with the specific mutation strategy as follows: aspartic acid (Asp) 294, glycine (Gly) 296, isoleucine (Ile) 312, and threonine (Thr) 328 were all mutated to arginine (Arg). The corresponding mutants were designated as D294R, G296R, I312R, and T328R, respectively. Co-IP assays demonstrated that the interaction between TRIM25 and the IGF2BP3 D294R mutant was significantly impaired, whereas the other mutations had no notable effect (Supplementary Fig. 4 C). Subsequent ubiquitination assays revealed that TRIM25-mediated ubiquitination was markedly reduced for the D294R mutant compared to wild-type IGF2BP3 (Supplementary Fig. 4D). Additionally, RIP experiments showed that the enrichment of LL22NC03 was significantly decreased with the IGF2BP3 D294R mutant (Supplementary Fig. 4E), indicating that Asp294 is essential for the interaction between IGF2BP3 and LL22NC03.

### LL22NC03 promotes HCC cell proliferation, migration, and invasion via IGF2BP3

To investigate the potential role of the interaction between IGF2BP3 and LL22NC03 in HCC progression, we conducted gain- and loss-of-function experiments in Huh7 and SK-HEP-1 cells. Cells were transfected with IGF2BP3 and/or LL22NC03 plasmids. Assessment of cell proliferation by CCK-8 and EdU assays revealed that LL22NC03 overexpression significantly enhanced HCC cell proliferation, whereas knockdown of IGF2BP3 markedly suppressed this proliferative effect (Supplementary Fig. 5A-B). Consistent with these findings, Transwell assays demonstrated that the enhanced migratory and invasive capacities induced by LL22NC03 overexpression were effectively reversed upon IGF2BP3 knockdown (Supplementary Fig. 5 C). We further validated these observations in patient-derived HCC organoid models. Overexpression of LL22NC03 promoted organoid growth, resulting in larger and more numerous organoids. Conversely, IGF2BP3 knockdown impaired organoid formation, yielding fewer and smaller organoids. Importantly, the pro-tumorigenic phenotype driven by LL22NC03 was significantly attenuated upon concurrent IGF2BP3 knockdown (Supplementary Fig. 5D-F). Collectively, these results indicate that the oncogenic function of LL22NC03 in HCC is dependent on IGF2BP3, highlighting a critical functional synergy between them in promoting HCC malignancy.

### LL22NC03/IGF2BP3 axis promotes glycolytic metabolism in HCC

**Fig. 4:**
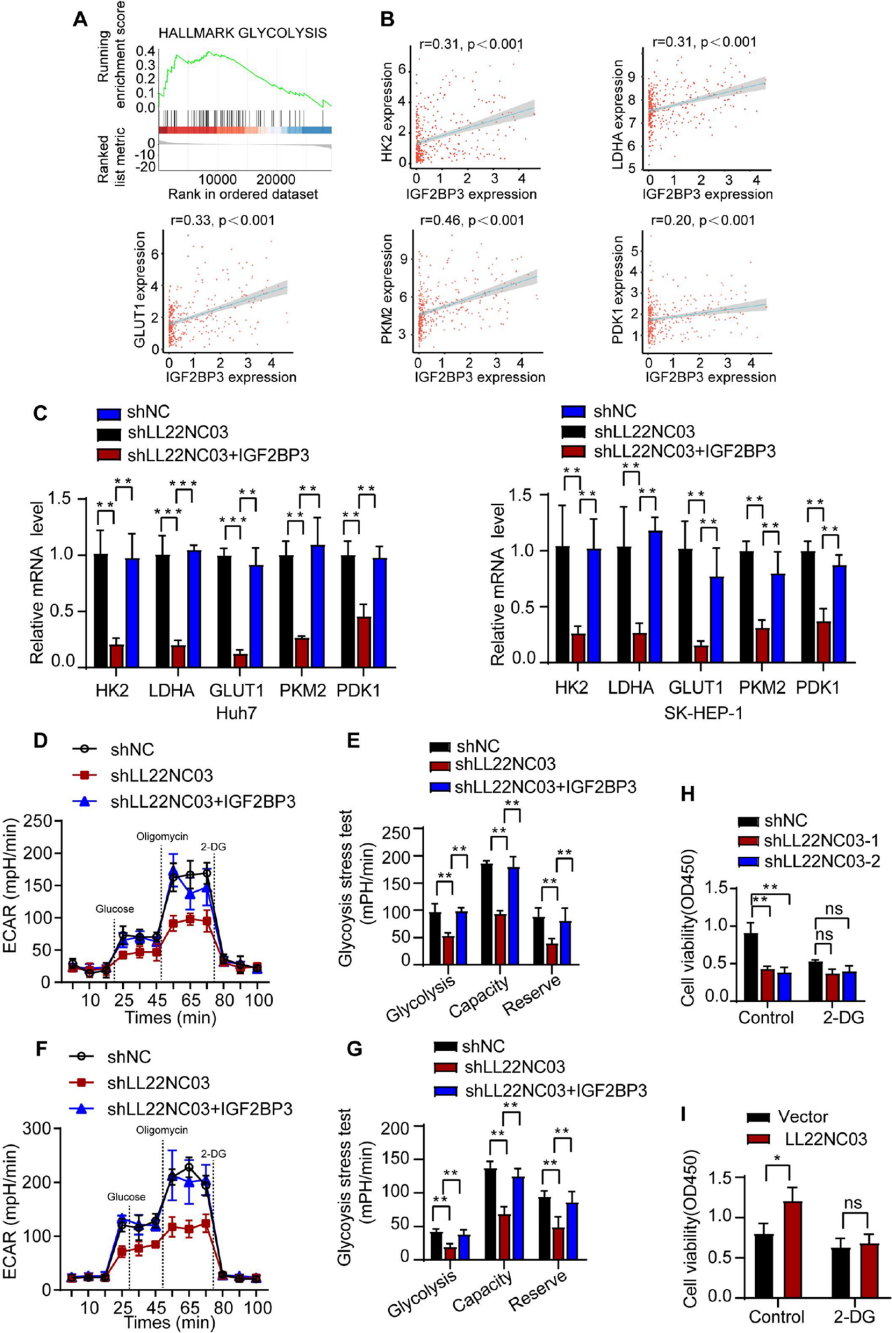
The LL22NC03/IGF2BP3 Axis Promotes Glycolytic Metabolism in HCC. **A** GSEA enrichment analysis revealed that IGF2BP3 overexpression significantly enhanced glycolytic activity in HCC cells. **B** Correlation of IGF2BP3 levels with HK2, LDHA, GLUT1, PKM2, and PDK1 mRNA levels in HCC (*n* = 351). The RNA levels were detected by q-PCR using GAPDH as an internal reference. **C** Overexpression of IGF2BP3 increased the mRNA levels of key genes involved in glycolysis (HK2, LDHA, GLUT1, PKM2, and PDK1) in LL22NC03-knockdown HCC cells. **D** and **E** Extracellular acidification rates were assayed using a Seahorse XF extracellular flow analyzer in shLL22NC03, shLL22NC03 + IGF2BP3, and control Huh7 cells. Glucose, oligomycin, and 2-DG were injected sequentially at different time points. shLL22NC03 resulted in a decrease in glycolytic capacity, and partial recovery of glycolytic capacity was observed with overexpression of IGF2BP3.** F** and **G** The rate of extracellular acidification was examined using a Seahorse XF extracellular flow analyzer in shLL22NC03, shLL22NC03 + IGF2BP3, and control SK-HEP-1 cells. Glucose, oligomycin, and 2-DG were injected sequentially at different time points. shLL22NC03 resulted in a decrease in glycolytic capacity, and partial restoration of glycolytic capacity was observed with IGF2BP3 overexpression. **H** LL22NC03-knockdown Huh7 cells (sh-1 and sh-2) were treated with or without the glycolysis inhibitor 2-DG (1 µM), and the effect on cell viability was determined via a CCK8 assay.** I** LL22NC03-overexpressing SK-HEP-1 cells were treated with or without the glycolysis inhibitor 2-DG (1 µM), and the effect on cell viability was determined via a CCK8 assay. (^*^P < 0.05, ^**^P < 0.01, ^***^P < 0.001, ns: not significant, *n* = 3)

Previous evidence has linked IGF2BP3 to the regulation of glycolytic genes and metabolic flux [27, 28]. To systematically evaluate its metabolic role in hepatocellular carcinoma, we first performed gene set enrichment analysis (GSEA), which revealed significant association between IGF2BP3 expression and glycolytic pathways (Fig. 4A). Further interrogation of TCGA-LIHC data demonstrated positive correlations between IGF2BP3 and key glycolytic enzymes including HK2, LDHA, GLUT1, PKM2, and PDK1 (Fig. 4B), suggesting IGF2BP3 as a potential regulator of glycolysis in HCC progression.

To establish the functional relationship between LL22NC03 and IGF2BP3 in glycolytic regulation, we performed loss-of-function and rescue experiments. qPCR analysis showed that LL22NC03 knockdown significantly downregulated glycolytic gene expression, which was effectively reversed by IGF2BP3 overexpression (Fig. 4C). ECAR measurements indicated that LL22NC03 depletion substantially impaired glycolytic activity in both Huh7 and SK-HEP-1 cells, as indicated by reduced glycolytic proton efflux, glycolytic capacity, and glycolytic reserve. Importantly, IGF2BP3 overexpression partially restored the glycolytic deficiency caused by LL22NC03 knockdown (Fig. 4D-G). To further confirm the functional significance of glycolytic metabolism in HCC proliferation, we treated cells with the glycolytic inhibitor 2-DG. This treatment resulted in marked suppression of cell proliferation (Fig. 4H, I). Collectively, our findings demonstrate that the LL22NC03/IGF2BP3 axis plays a critical role in maintaining glycolytic metabolism, thereby promoting malignant progression in HCC.

### LL22NC03 regulates MYC expression stability via IGF2BP3 in an m6A-dependent manner

The MYC proto-oncogene plays a pivotal role in cell growth, metabolic reprogramming, and tumorigenesis, with its aberrant expression being closely associated with progression of hepatocellular carcinoma and other malignancies. Previous studies have established MYC as an important downstream target of IGF2BP3 [29]. To investigate the functional relationship between MYC and the previously identified LL22NC03/IGF2BP3 axis, we first performed bioinformatic analyses using TCGA-LIHC data. These revealed significant positive correlations between MYC expression and both IGF2BP3 and key glycolytic genes, including HK2, LDHA, GLUT1, PKM, and PDK1 (Supplementary Figs. 6 A,B), suggesting MYC’s potential involvement in the IGF2BP3-mediated metabolic regulatory network.

**Fig. 5:**
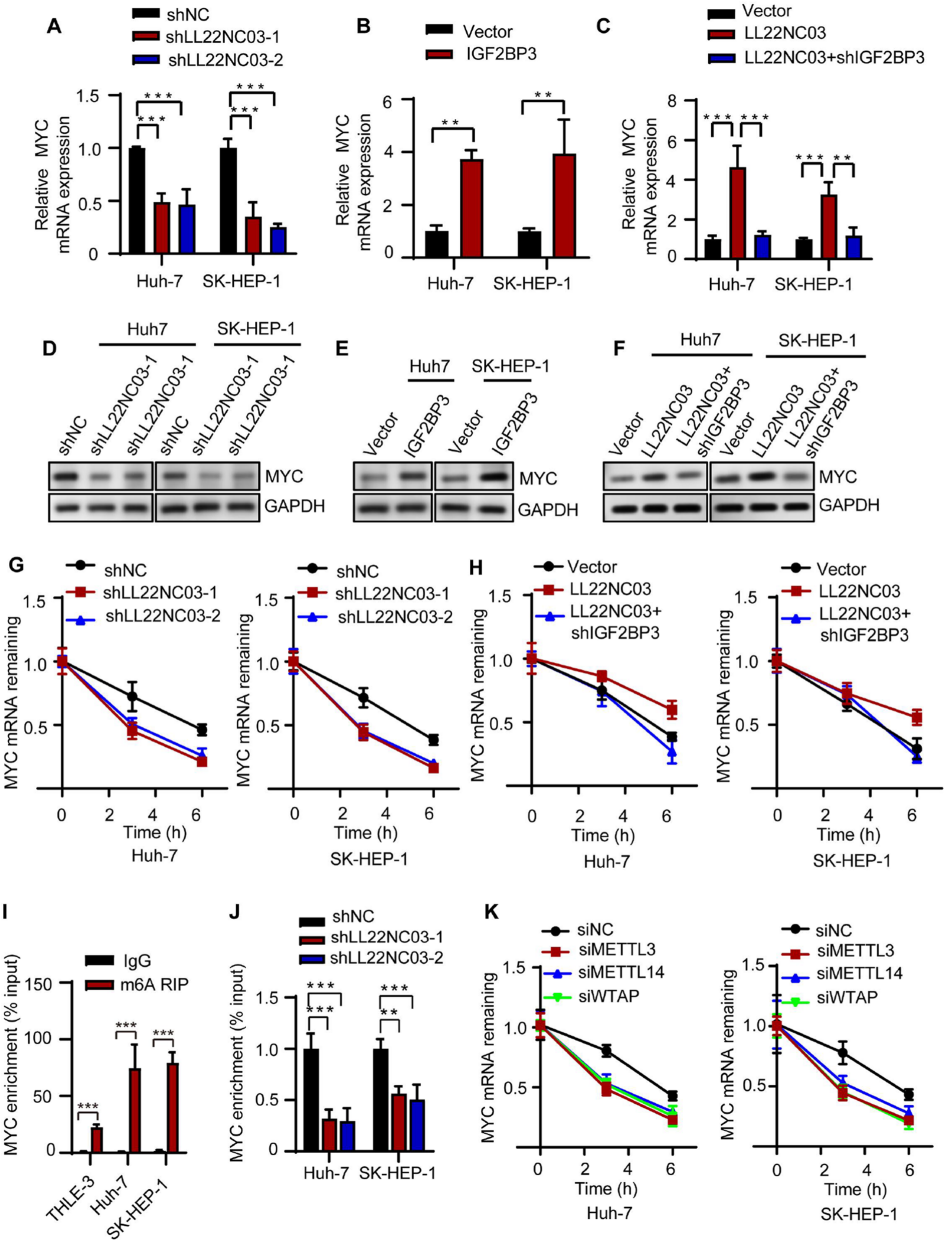
LL22NC03 Regulates MYC Expression Stability via IGF2BP3 in an m6A-Dependent Manner. **A** The mRNA expression level of MYC was decreased in Huh7 and SK-HEP-1 cells with LL22NC03-knockdown. **B** The mRNA expression level of MYC was elevated in Huh7 and SK-HEP-1 cells overexpressing IGF2BP3. **C** Knockdown of IGF2BP3 inhibited MYC mRNA expression in LL22NC03-overexpressing HCC cells. **D** The protein expression levels of MYC were decreased in Huh7 and SK-HEP-1 cells after LL22NC03-knockdown. **E** The protein expression levels of MYC were elevated in Huh7 and SK-HEP-1 cells after IGF2BP3 overexpression.** F** Knockdown of IGF2BP3 inhibited MYC protein expression in LL22NC03-overexpressing HCC cells. **G** The stability of MYC in Huh7 and SK-HEP-1 cells was examined by actinomycin D treatment. The stability of MYC mRNA decreased when LL22NC03 was knocked down (sh-1 and sh-2). **H** In cells treated with actinomycin D, knocking down IGF2BP3 can partially reverse the increase in MYC mRNA stability caused by overexpression of LL22NC03. **I** Detection of MYC endogenous m6A modification background levels in normal and HCC cell lines. **J** LL22NC03 knockdown in Huh7 and SK-HEP-1 cells (sh-1 and sh-2) reduced IGF2BP3-enriched MYC mRNA levels. **K** Silencing of m6A-modifying enzymes (METTL3, METTL14, and WTAP) decreased MYC mRNA stability in both Huh7 and SK-HEP-1 cells. (^**^P < 0.01, ^***^P < 0.001, *n* = 3)

At the cellular level, knockdown of LL22NC03 significantly reduced MYC mRNA expression in hepatocellular carcinoma cells (Fig. 5A), whereas overexpression of IGF2BP3 effectively upregulated MYC mRNA levels (Fig. 5B). Notably, in the context of LL22NC03 overexpression, depletion of IGF2BP3 rescued the enhanced MYC expression induced by LL22NC03 (Fig. 5C). Furthermore, Western blot analysis and mRNA stability assays consistently demonstrated parallel effects at both the protein level and transcript stability (Fig. 5D–H), indicating that IGF2BP3 acts as a key mediator in the LL22NC03-dependent regulation of MYC. Given that IGF2BPs have been identified as specific m6A readers that enhance mRNA stability through recognition of m6A-modified transcripts [29], we further investigated this regulatory mechanism in HCC. Our experimental results showed significantly elevated m6A modification levels on MYC mRNA in HCC cells (Fig. 5I). LL22NC03 knockdown markedly reduced m6A enrichment on MYC mRNA (Fig. 5J). Mechanistically, siRNA-mediated knockdown of m6A methyltransferase complex components (METTL3, METTL14, or WTAP) accelerated MYC mRNA decay (Fig. 5K), demonstrating the crucial role of m6A modification in maintaining MYC mRNA stability. In summary, our study reveals a molecular pathway through which LL22NC03 regulates MYC expression levels via IGF2BP3-mediated m6A modification-dependent stabilization. Importantly, our data do not imply universal regulation of MYC by all M6A writers; rather, the effect appears context-dependent and limited to the components tested here.

### IGF2BP3 promotes glycolytic metabolism and malignant progression of HCC through MYC

**Fig. 6:**
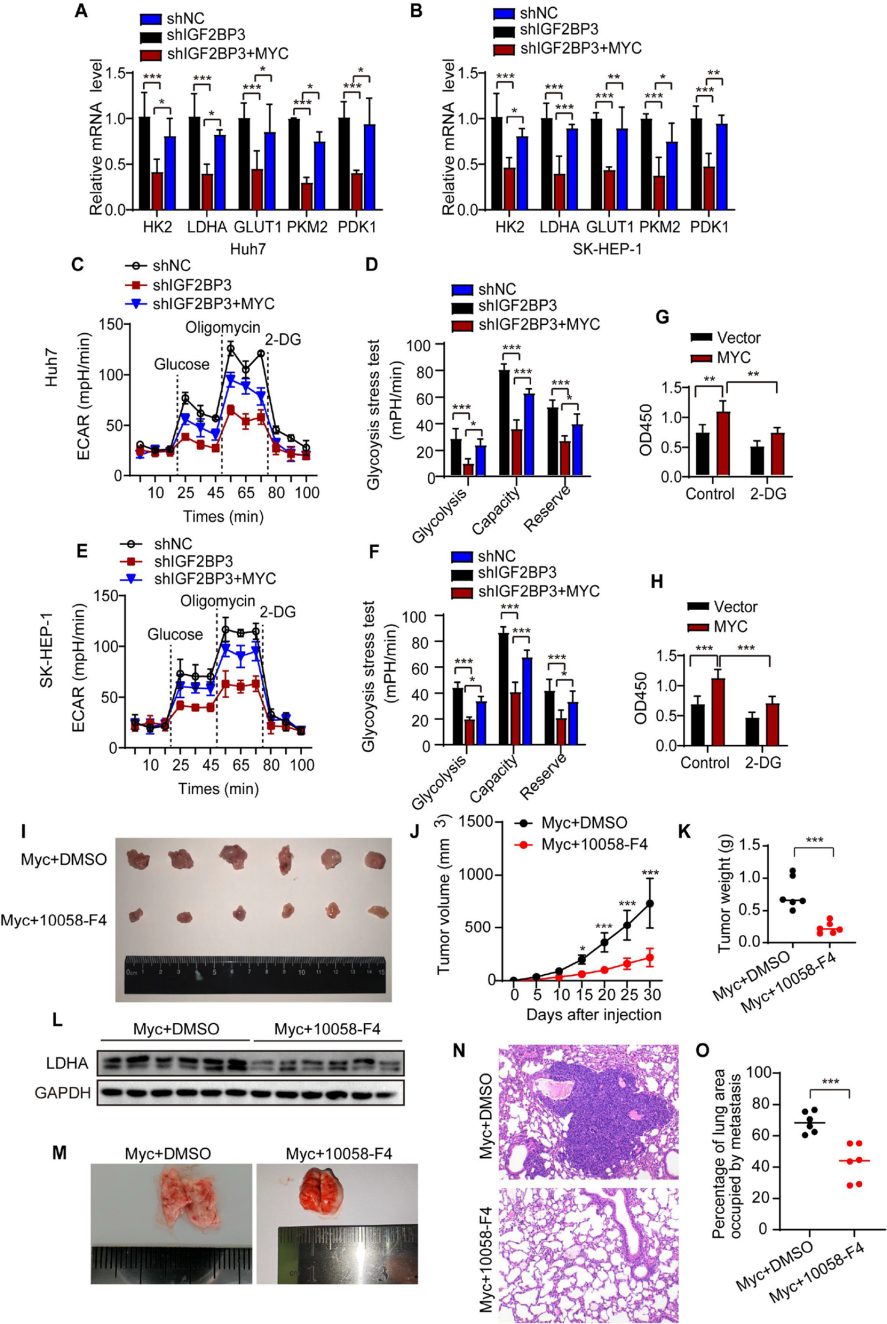
IGF2BP3 Promotes Glycolytic Metabolism and Malignant Progression of HCC through MYC. **A** and **B** Overexpression of MYC increased the mRNA levels of key genes involved in glycolysis (HK2, LDHA, GLUT1, PKM2, and PDK1) in IGF2BP3-knockdown HCC cells. **C-F** Extracellular acidification rates were assayed using a Seahorse XF extracellular flow analyzer in shIGF2BP3, shIGF2BP3 + MYC, and control Huh7 and SK-HEP-1 cells. Glucose, oligomycin, and 2-DG were injected sequentially at different time points. shIGF2BP3 resulted in a decrease in glycolytic capacity, and partial recovery of glycolytic capacity was observed with overexpression of MYC. **G** and **H** MYC-overexpressing Huh7 and SK-HEP-1 cells were treated with or without the glycolysis inhibitor 2-DG (1 µM), and measure cell survival rate using CCK8 assay. **I**. Representative images of tumor sizes in mice following subcutaneous injection of Myc-overexpressing human liver cancer organoids, with or without the administration of 10058-F4 (*n* = 6). **J** Growth curves of tumor volume in mice. **K** Tumor weights in mice used for the subcutaneous tumor model. **L** Western Blot analysis of LDHA protein levels in the transplanted tumors. **M** Representative images showing the impact of administering or not administering 10058-F4 on lung metastasis in mice after tail vein injection of Myc-overexpressing human liver cancer organoids (10 × magnification). **N** Representative images of HE-stained lung metastases in tumor sections from the tail vein tumor model. **O** Percentage of lung area occupied by metastases in the tail vein tumor model. (*P < 0.05, **P < 0.01, ***P < 0.001, ns: not significant)

To further investigate the role of MYC in the IGF2BP3-mediated glycolytic pathway, we systematically evaluated changes in the expression of glycolytic genes and metabolic activity. In Huh7 and SK-HEP-1 cells, knockdown of IGF2BP3 significantly reduced the mRNA levels of key glycolytic genes, including HK2, LDHA, GLUT1, PKM2, and PDK1, whereas MYC overexpression partially reversed this suppression (Fig. 6A, B), suggesting that MYC acts as a critical downstream mediator of IGF2BP3 in regulating glycolytic gene expression. ECAR assays demonstrated that IGF2BP3 knockdown markedly decreased glycolytic flux, which was partially restored by MYC supplementation. Glycolytic stress tests further confirmed that the impairment in glycolytic capacity and reserve induced by IGF2BP3 depletion could be partially rescued by MYC overexpression (Fig. 6C–F). Additionally, treatment with the glycolytic inhibitor 2-DG effectively suppressed the proliferation advantage of MYC-overexpressing cells (Fig. 6G, H), indicating that MYC-driven proliferation depends on functional glycolytic metabolism.

To evaluate the therapeutic potential of targeting MYC in vivo, we established a xenograft model by subcutaneously injecting MYC-overexpressing human HCC organoids into mice. The mice were divided into a DMSO control group and a 10058-F4 (MYC inhibitor) treatment group. Tumor volume and weight in the inhibitor-treated group were significantly smaller than those in the control group (Fig. 6I). Tumor growth curves (Fig. 6J) and weight measurements (Fig. 6K) further confirmed that MYC inhibition significantly delayed tumor growth. Western blot analysis revealed a pronounced downregulation of LDHA protein expression after inhibitor treatment (Fig. 6L), indicating that MYC blockade effectively suppresses tumor glycolytic activity. A metastasis model was established via tail vein injection to assess the role of MYC in HCC metastasis. Macroscopic examination showed that the number and size of lung metastatic nodules were significantly reduced in the inhibitor-treated group (Fig. 6M). H&E staining of lung tissues further confirmed extensive pulmonary metastases in the control group, whereas MYC inhibition significantly alleviated the metastatic burden (Fig. 6N, O), demonstrating the critical role of MYC in promoting HCC metastasis. These findings indicate that IGF2BP3 regulates glycolytic metabolism and promotes the malignant progression of HCC through MYC.

### The LL22NC03-IGF2BP3-MYC axis promotes HCC Growth and metastasis by regulating glycolysis

**Fig. 7:**
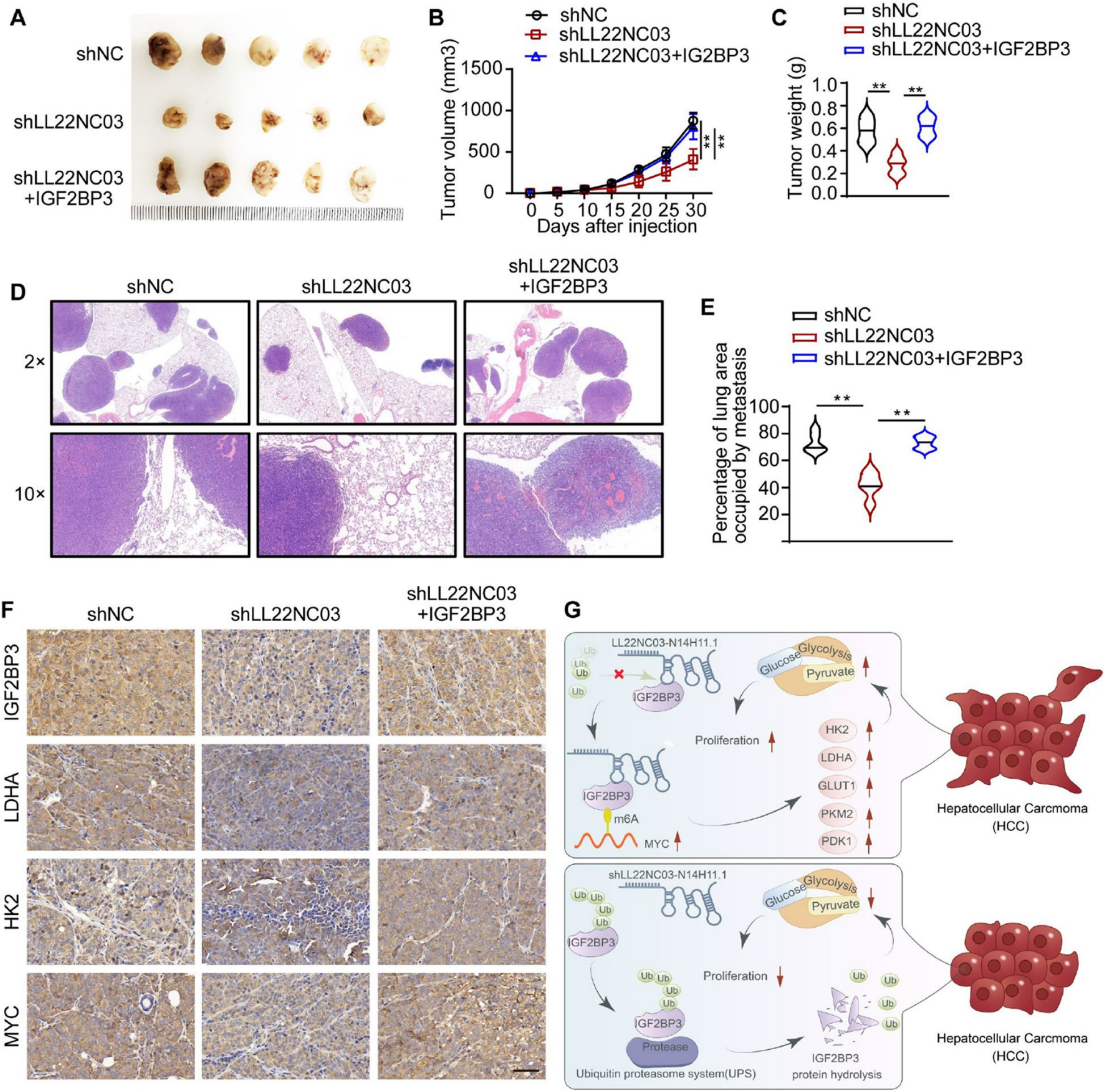
The LL22NC03–IGF2BP3–MYC Axis Promotes HCC Growth and Metastasis by Regulating Glycolysis. **A** The in situ tumors were subcutaneously excised from the nude mice used in the subcutaneous tumor model. **B** Growth curves of tumor volume in the mice. **C** Tumor weights of the mice used in the subcutaneous tumor model. **D** Representative images of HE-stained lung metastases in tumor sections from the caudal vein graft tumor model. **E** Percentage of the lung area occupied by lung metastases in tumors in the tail vein tumor model. **F** Representative images of IHC staining for IGF2BP3, LDHA, KH2, and MYC were obtained from tumor sections. Scale bar = 50 μm. **G** A working model was proposed for this study. LL22NC03 stabilized IGF2BP3 by binding to the ubiquitination site of IGF2BP3, which in turn maintained MYC-induced glycolysis and proliferation of HCC cells. Conversely, inhibition of the LL22NC03 resulted in increased degradation of IGF2BP3 through the ubiquitination pathway. (^**^P < 0.01, *n* = 5)

To validate the role of the LL22NC03-IGF2BP3-MYC axis in HCC progression, we established stable Huh7 cell lines with LL22NC03 knockdown, either alone or in combination with IGF2BP3 overexpression, and subcutaneously injected them into BALB/c nude mice to generate xenograft models. The results demonstrated that IGF2BP3 overexpression significantly rescued the tumor growth inhibition caused by LL22NC03 knockdown, as reflected by increased tumor volume and weight (Figs. 7A–C). To further assess the impact of this regulatory axis on metastasis, a lung metastasis model was established via tail vein injection. H&E staining of lung tissues revealed that LL22NC03 knockdown markedly reduced the metastatic burden, while IGF2BP3 overexpression partially restored metastatic capacity under LL22NC03-knockdown conditions (Fig. 7D, E). IHC analysis of subcutaneous xenograft tissues showed that LL22NC03 knockdown significantly downregulated the expression of IGF2BP3, MYC, and the glycolysis-related proteins LDHA and HK2. Conversely, IGF2BP3 overexpression effectively reversed the LL22NC03 knockdown–induced suppression of MYC, LDHA, and HK2 expression (Fig. 7F). In summary, our findings demonstrate that LL22NC03 stabilizes IGF2BP3, thereby activating the MYC-mediated glycolytic pathway, which ultimately drives HCC growth and metastasis (Fig. 7G).

## Conclusion

Our study elucidates the critical role of the LL22NC03-IGF2BP3-MYC axis in driving HCC progression. We demonstrate that LL22NC03 acts as an oncogenic lncRNA by binding to IGF2BP3, shielding it from TRIM25-mediated ubiquitination and subsequent degradation. This stabilization of IGF2BP3 enhances its ability to promote MYC transcription, which in turn upregulates key glycolytic enzymes (HK2, LDHA, GLUT1, PKM2, and PDK1), sustaining HCC cell proliferation, migration, and invasion. Furthermore, our findings highlight the m6A-dependent stabilization of MYC by IGF2BP3, adding another layer to the regulatory network linking RNA metabolism to HCC pathogenesis. Given the central role of this axis in HCC progression, targeting LL22NC03 or the IGF2BP3-MYC interaction may offer a promising therapeutic strategy.

## Discussion

LncRNAs play pivotal roles in cellular differentiation and development, and their dysregulation is closely associated with cancer onset [30]. Novel lncRNAs with unique functional mechanisms continue to be identified in HCC [31]. For example, the LINC00326-CCT3 axis influences HCC development and progression through lipid metabolism regulation [32]. Similarly, the IGF2BP2-lncRNA TRPC7-AS1 axis impacts HMGA2 expression, which promotes HCC progression [32]. LINC00998 encodes the conserved peptide SMIM30, which facilitates HCC progression by promoting SRC/YES1 membrane anchoring and activating the MAPK pathway [33]. Additionally, SNHG6 regulates cholesterol metabolism and drives HCC progression [34]. These findings underscore the necessity for further research into the roles of lncRNAs in HCC.

Our previous research revealed that the highly expressed oncogenic lncRNA LL22NC03 is correlated with poor prognosis in HCC patients [8]. By examining the regulatory impacts of LL22NC03 on HCC progression and mitochondrial fission, our investigation revealed that LL22NC03 promotes HCC progression by stimulating mitochondrial fission via activation of the H-RAS/MAPK signaling pathway [8]. Given the paucity of literature regarding the functionality and molecular mechanisms of LL22NC03, our research seeks to investigate these aspects in greater depth. Our findings indicate that the overexpression of LL22NC03 markedly enhances the proliferation, invasiveness, and migration of HCC cells, whereas its downregulation inhibits these cellular processes.

The interaction between IGF2BP3 and specific mRNAs influences the expression of cancer-related genes, which impacts cell proliferation, migration, and survival. Protein interaction, coupled with mass spectrometry analysis, revealed a direct interaction between the LL22NC03 and IGF2BP3. Recent studies suggest that ubiquitin E3 ligase HECTD4 [35], Parkin [36], TRIM21 [37] and TRIM25 [20, 21] modulate the expression of IGF2BP3 through the ubiquitin pathway. In the present study, we found that TRIM25 not only inversely regulated the expression of the IGF2BP3 protein, but also interacted with IGF2BP3 in HCC cells. Moreover, TRIM25 significantly promoted K63- but not K48-linked ubiquitination of IGF2BP3. The Co-IP assay indicated that LL22NC03 reduced the connection between TRIM25 and IGF2BP3 and the overexpression of LL22NC03 could rescue the downregulation of IGF2BP3 caused by TRIM25.

Glycolysis plays a crucial role in facilitating tumor cell proliferation, migration, and invasion, even in oxygen-rich conditions [38–41]. Dysregulated glycolysis is a key feature of metabolic reprogramming, which contributes to HCC tumorigenesis in experimental models [42]. Research has established a link between glycolysis and lncRNAs in tumor progression. However, the precise mechanisms by which lncRNAs regulate glycolysis in HCC was not completely understood. IGF2BP3, as a member of RBP, has been known to stabilize mRNA of downstream target genes [43]. m6A methylation affects the RNA stability of LDHA by IGF2BP3 [44, 45]. IGF2BP3 is an m6A reader essential in various malignancies [46]. The aberrantly high expression of IGF2BP3 can preferentially detect m6A-modified target RNAs, thereby facilitating target RNA stability and expression, causing glycolysis [47]. circFOXK2 promoted the GLUT1 mRNA stability through cooperating with IGF2BP3 in a m^6^A-dependent manner [47]. IGF2BP3 can stabilize the mRNA of PKM2 [48, 49]. In this study, we demonstrated that the mRNA expression levels of glycolysis-related genes (HK2, LDHA, GLUT1, PKM2, PDK1) were positively correlated with the IGF2BP3 level in HCC tissues. Moreover, impaired glycolysis resulting from LL22NC03 knockdown was partially restored by IGF2BP3 overexpression in HCC cells. GSEA revealed a strong correlation between IGF2BP3 and glycolysis. Treatment with the glycolysis inhibitor 2-DG reduced the proliferation of HCC cells with elevated LL22NC03 expression.

MYC is reported as an oncogene and promotes tumor proliferation by increasing the glycolytic activity of cancer cells under normoxic conditions [50]. It has been well-documented that MYC promotes glycolysis through transcriptionally upregulating GLUT1, HK2, PKM2 and LDHA [51–54]. Given that MYC mRNA is a well-known target of IGF2BP3 and a key regulator of glycolysis, our study examined MYC expression and revealed interactions among LL22NC03, IGF2BP3, and MYC. These results also demonstrated a positive correlation between MYC mRNA and protein levels and the expression of LL22NC03 and IGF2BP3. The knockdown of IGF2BP3 in HCC cells, which overexpressed LL22NC03, led to a reduction in MYC mRNA and protein levels, along with diminished protein stability. This suggests the presence of compensatory mechanisms. Our study revealed significantly higher endogenous m6A modification levels in HCC cell lines than in normal cell lines. Silencing m6A-modifying enzymes (METTL3, METTL14 and WTAP) led to decreased MYC mRNA stability across all cell lines tested, which indicates a potential role of the methylation pathway in regulating MYC mRNA stability. Additionally, our findings suggest that IGF2BP3 may maintain mRNA stability by recognizing m6A-modified mRNAs and recruiting RNA stabilizers. This highlights a regulatory network involving LL22NC03, IGF2BP3 and MYC that bridges lncRNA epigenetic networks with m6A modifications.

Beyond mechanistic insight, our data nominate LL22NC03 as a potential therapeutic entry point in HCC. Practical modalities include antisense oligonucleotides (e.g., LNA/GapmeR; with GalNAc conjugation to enhance hepatocyte uptake), siRNA (lipid nanoparticles or GalNAc conjugates), and CRISPR interference for locus-specific repression. Selection among these approaches should consider subcellular localization and on-target engagement of the lncRNA, as well as delivery, durability, and safety. While a head-to-head therapeutic evaluation is beyond the current scope, these avenues provide a concrete roadmap for future preclinical development.

Several limitations should be acknowledged. First, the clinical relevance of LL22NC03 was primarily assessed using TCGA data, and further validation in independent, multi-ethnic cohorts with larger sample sizes is needed to confirm its prognostic and therapeutic significance. Second, the study focused on the glycolytic pathway regulated by MYC, but other potential downstream effectors of LL22NC03-IGF2BP3 signaling remain unexplored, leaving room for broader mechanistic investigations.

## Conclusions

Our work identifies and characterizes the oncogenic LL22NC03-IGF2BP3-MYC regulatory axis that promotes HCC progression by enhancing glycolytic metabolism, providing new insights into HCC pathogenesis and potential therapeutic targets.

## Supplementary Information

Supplementary Material 1: Supplementary Figure 1 LL22NC03 is upregulated in HCC tissues and promotes proliferation, migration and invasion of HCC cells. A. qRT-PCR analysis of LL22NC03 expression differences between 10 pairs of HCC tissues and adjacent non-tumor tissues. B. qRT-PCR validation of LL22NC03 overexpression efficiency in Huh7 and SK-HEP-1 cell lines. C. CCK-8 assay for proliferation rates of Huh7 and SK-HEP-1 cells. Compared with the control group, the cell proliferation ability was significantly enhanced in the LL22NC03 overexpression group. D. EdU assay for proliferation ability of HCC cells (EdU-positive cells: red; DAPI: blue). Compared with the control group, the proportion of EdU-positive cells was significantly increased in the LL22NC03 overexpression group. Scale bar = 50 μm. E. Transwell assay for migration and invasion abilities of HCC cells. Compared with the control group, the number of migrated and invaded cells was significantly increased in the LL22NC03 overexpression group. Scale bar = 100 μm. F. Wound healing assay for migration ability of HCC cells. Compared with the control group, the migration distance was significantly increased in the LL22NC03 overexpression group. Scale bar = 100 μm. (^*^P<0.05,^**^P<0.01, *n*=3). Supplementary Figure2 Structural and phenotypic characteristics of native tumor tissue and HCC organoids. A. Hematoxylin and Eosin (H&E) staining of primary HCC tumors and their derived organoids. Scale bar = 10 μm and 100 μm. B. Immunofluorescence staining was performed to detect Epcam expression in primary HCC tumors and their derived organoids (Epcam: green; DAPI: blue). Scale bar = 20 μm and 100 μm. Supplementary Figure 3A. qPCR analysis of IGF2BP3 expression in 10 paired HCC clinical samples. (**p < 0.01). Supplementary Figure 4 Mapping of the interaction domains between TRIM25 and IGF2BP3 and validation of the critical binding residue. A. LIGPLOT-generated"eyelash plot" for TRIM25-IGF2BP3 interaction. B. Electrostatic potential map of TRIM25-IGF2BP3 complex constructed by PyMOL. C. Co-IP assay for TRIM25 interaction with IGF2BP3 wild-type (WT) or mutants (D294R, G296R, I312R, T328R). D. Co-IP assay of ubiquitination levels of IGF2BP3 WT and D294R mutant mediated by TRIM25. E. RIP assay for LL22NC03 enrichment on IGF2BP3 WT or D294R mutant. (**P<0.01, ns: not significant; *n*=3). Supplementary Figure 5 LL22NC03 promotes the proliferation, migration, and invasion of HCC cells via IGF2BP3. A and B. CCK-8 and EdU assays demonstrated that LL22NC03 overexpression significantly promoted cell proliferation in Huh7 and SK-HEP-1 cells, an effect that was suppressed by IGF2BP3 knockdown. C. Transwell assays revealed that IGF2BP3 knockdown reversed the enhanced migratory and invasive capacities induced by LL22NC03 overexpression in Huh7 and SK-HEP-1 cells. D. Representative images of HCC organoids from each experimental group. Scale bar = 100 µm. E. Quantitative analysis of total proliferative area in HCC organoids. IGF2BP3 knockdown attenuated the LL22NC03-driven increase in total organoid area. F. Quantitative analysis of HCC organoid formation efficiency. IGF2BP3 knockdown significantly reduced the enhanced organoid formation efficiency promoted by LL22NC03 overexpression. (*P < 0.05, **P < 0.01, ns: not significant; *n* = 3). Supplementary Figure 6 Correlation between MYC expression and key glycolytic genes in hepatocellular carcinoma (TCGA-LIHC) samples. A. The correlation between IGF2BP3 and MYC expression (*n*=351). B. The correlation between HK2, LDHA, SLC2A1, PKM, and PDK1 gene expression and MYC expression (*n*=351).

## Data Availability

The data used to support the findings of this study are available from the corresponding author upon request.

## Abbreviations

HCC: Hepatocellular carcinoma
lncRNA: Long non-coding RNA
IGF2BP3: Insulin-like growth factor 2 mRNA-binding protein 3
m6A: N6-methyladenosine
MYC: MYC proto-oncogene, bHLH transcription factor
LDHA: Lactate dehydrogenase A
GLUT1: Glucose transporter 1 (SLC2A1)
RIP: RNA Immunoprecipitation
Co-IP: Co-immunoprecipitation
CHX: Cycloheximide
MG132: Proteasome inhibitor MG132
RBP: RNA-binding protein
TCGA: The Cancer Genome Atlas
GSEA: Gene Set Enrichment Analysis
qRT-PCR: Quantitative real-time polymerase chain reaction
IHC: Immunohistochemistry
