# Supplementary material for: LL22NC03-N14H11.1 regulates the m6A modification of MYC and promotes glycolytic activity in hepatocellular carcinoma cells by inhibiting IGF2BP3 ubiquitination degradation
Source: J Exp Clin Cancer Res. 2025 Dec 2;45:14. doi: 10.1186/s13046-025-03606-1 (PMC12817445; doi:10.1186/s13046-025-03606-1)

Supplementary Figure 1

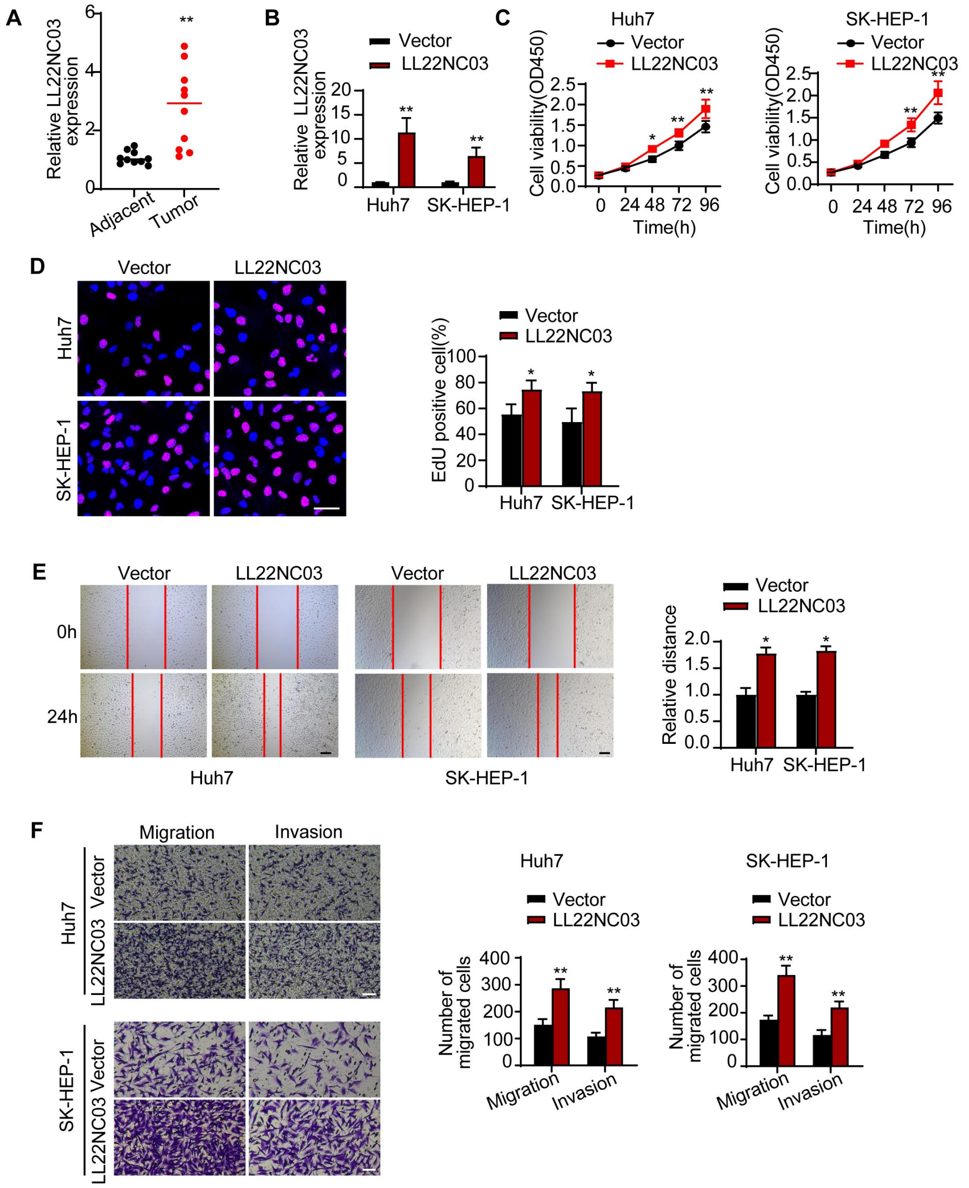

Supplementary Figure 2

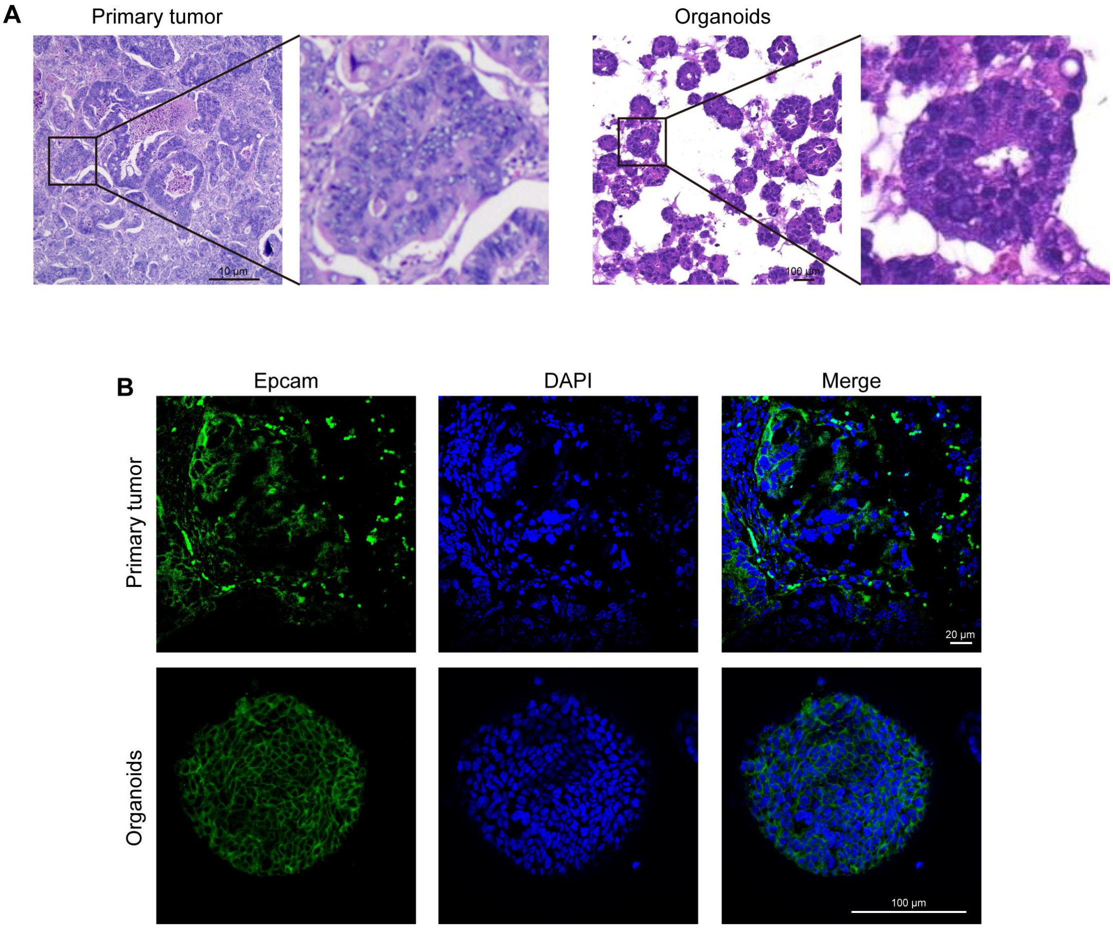

Supplementary Figure 3

A

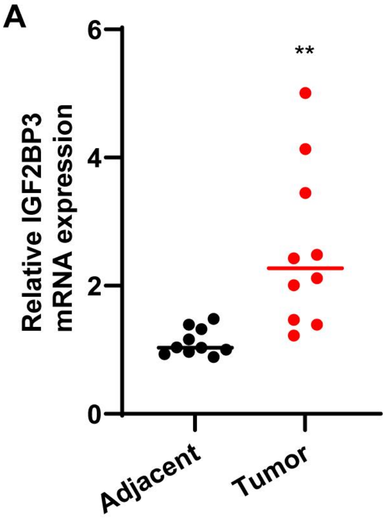

Supplementary Figure 4

A

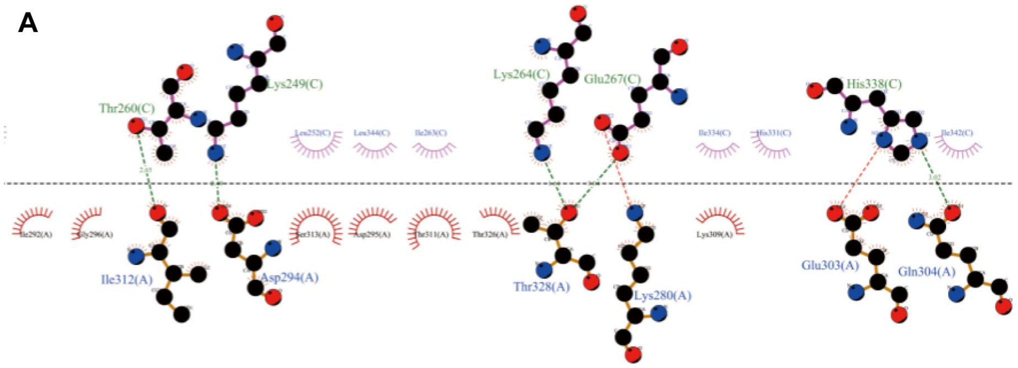

B

Binding energy	Hydrogen bonds (IGF2BP3:TRIM25)	Salt bridges
-6.6kcal/mol	GLY 296:GLU 256<br>IlE 312:THR 260<br>THR 328:GLU 267<br>ASP 294:LYS 249	LYS 280:GLU 267<br>ASP 294:LYS 249<br>GLU 303:HIS 338

C

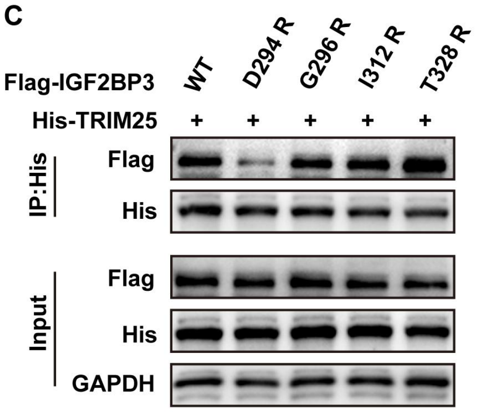

D

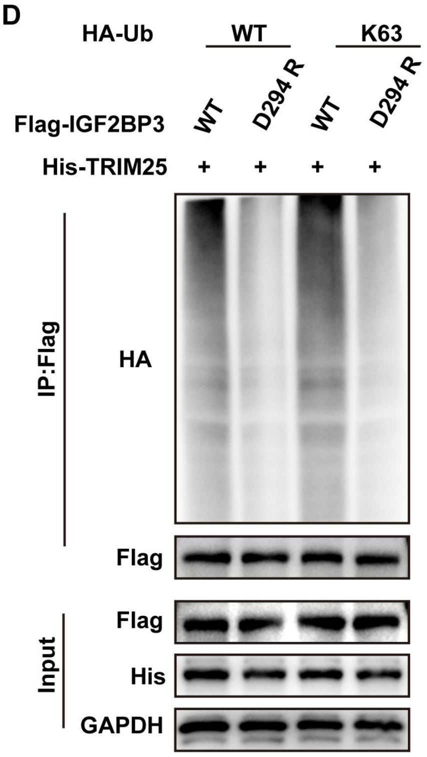

E

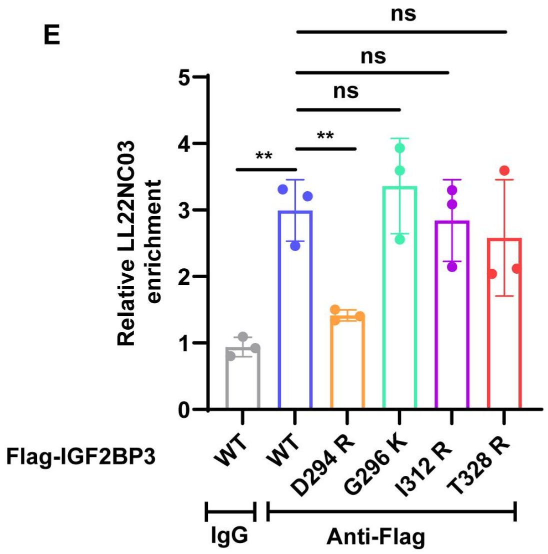

Supplementary Figure 5

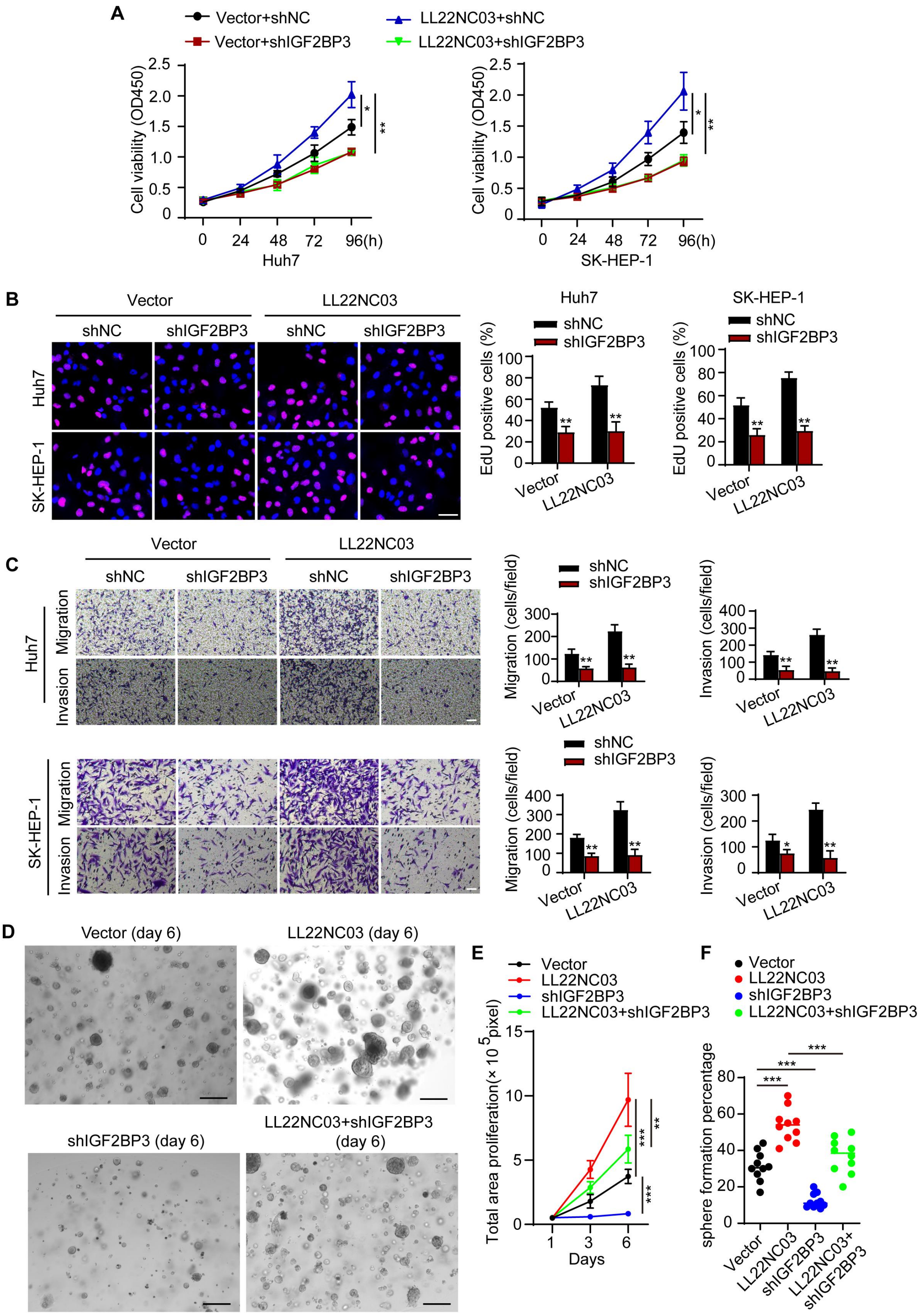

Supplementary Figure 6

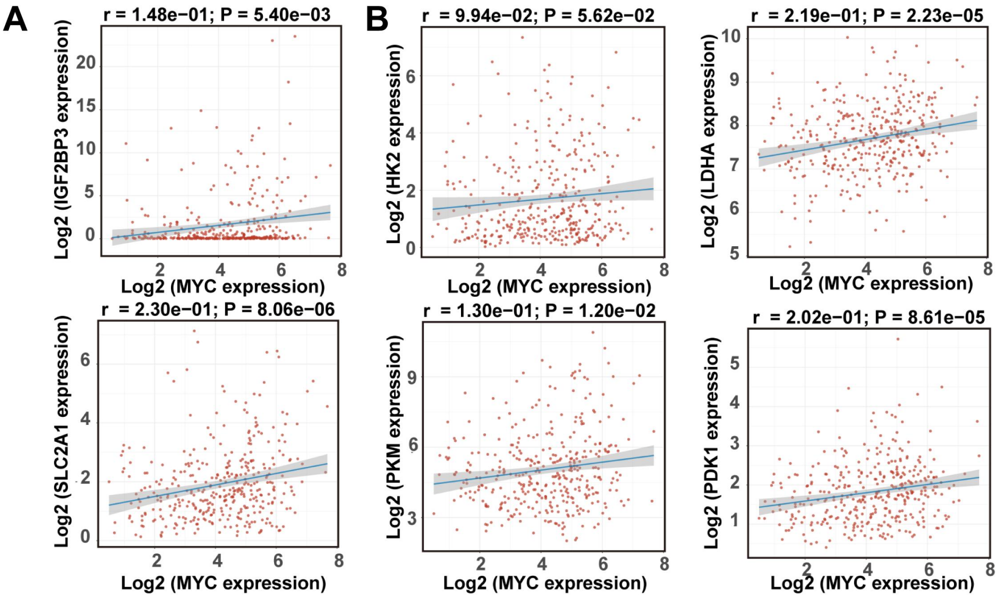

Supplement: Supplementary file 1 — Supplementary Material 1: Supplementary Figure 1 LL22NC03 is upregulated in HCC tissues and promotes proliferation, migration and invasion of HCC cells. A. qRT-PCR analysis of LL22NC03 expression differences between 10 pairs of HCC tissues and adjacent non-tumor tissues. B. qRT-PCR validation of LL22NC03 overexpression efficiency in Huh7 and SK-HEP-1 cell lines. C. CCK-8 assay for proliferation rates of Huh7 and SK-HEP-1 cells. Compared with the control group, the cell proliferation ability was significantly enhanced in the LL22NC03 overexpression group. D. EdU assay for proliferation ability of HCC cells (EdU-positive cells: red; DAPI: blue). Compared with the control group, the proportion of EdU-positive cells was significantly increased in the LL22NC03 overexpression group. Scale bar = 50 μm. E. Transwell assay for migration and invasion abilities of HCC cells. Compared with the control group, the number of migrated and invaded cells was significantly increased in the LL22NC03 overexpression group. Scale bar = 100 μm. F. Wound healing assay for migration ability of HCC cells. Compared with the control group, the migration distance was significantly increased in the LL22NC03 overexpression group. Scale bar = 100 μm. (*P<0.05,**P<0.01, n=3). Supplementary Figure2 Structural and phenotypic characteristics of native tumor tissue and HCC organoids. A. Hematoxylin and Eosin (H&E) staining of primary HCC tumors and their derived organoids. Scale bar = 10 μm and 100 μm. B. Immunofluorescence staining was performed to detect Epcam expression in primary HCC tumors and their derived organoids (Epcam: green; DAPI: blue). Scale bar = 20 μm and 100 μm. Supplementary Figure 3A. qPCR analysis of IGF2BP3 expression in 10 paired HCC clinical samples. (**p < 0.01). Supplementary Figure 4 Mapping of the interaction domains between TRIM25 and IGF2BP3 and validation of the critical binding residue. A. LIGPLOT-generated"eyelash plot" for TRIM25-IGF2BP3 interaction. B. Electrostatic pot [file 13046_2025_3606_MOESM1_ESM.pdf]
